# Genetically predicted basal metabolic rate and venous thromboembolism risk: a Mendelian randomization study

**DOI:** 10.3389/fnut.2023.1263804

**Published:** 2023-12-21

**Authors:** Jian Huang, Yubo Xie

**Affiliations:** ^1^Clinical Laboratory Center, The First Affiliated Hospital of Guangxi Medical University, Nanning, China; ^2^Department of Anesthesiology, The First Affiliated Hospital of Guangxi Medical University, Nanning, China; ^3^Guangxi Key Laboratory of Enhanced Recovery After Surgery for Gastrointestinal Cancer, The First Affiliated Hospital of Guangxi Medical University, Nanning, China

**Keywords:** basal metabolic rate, venous thromboembolism, deep vein thrombosis of lower extremities, pulmonary embolism, Mendelian randomization

## Abstract

**Background:**

Basal metabolic rate (BMR) is the minimum amount of energy needed by the body to carry out essential physiological functions. The goal of this study was to evaluate whether BMR causally influences venous thromboembolism (VTE) and its subtypes in European individuals.

**Methods:**

A two-sample Mendelian randomization (MR) was performed. Within a genome-wide association study (GWAS) involving 454,874 people, genetic variants were chosen as instrumental variables based on their significant associations (*p* < 5 × 10^−8^) with BMR and their limited linkage disequilibrium (*r*^2^ < 0.001). The FinnGen project served as sources for summary statistics of VTE, encompassing different subtypes.

**Results:**

Using the multiplicative random-effect inverse variance weighted method, our investigation revealed that one standard deviation higher BMR was associated with VTE (odds ratio [OR] = 1.684, 95% confidence interval [CI]: 1.465–1.936, *p* = 2.339 × 10^−13^), PE (OR = 1.824, 95% CI: 1.512–2.200, *p* = 3.399 × 10^−10^), and DVT of lower extremities (OR = 1.887, 95% CI: 1.562–2.280, *p* = 4.778 × 10^−11^). The consistency of these associations was observed in sensitivity analyses using various MR techniques like Mendelian randomization pleiotropy residual sum and outlier, MR-Egger, weighted median, and contamination mixture method. In addition, multivariable MR revealed direct effects of BMR on VTE and its subtypes when taking body mass index and current tobacco smoking into account.

**Conclusion:**

Higher BMR may increase the risk of VTE and its subtypes including PE and DVT of lower extremities.

## Introduction

Venous thromboembolism (VTE), including pulmonary embolism (PE) and deep vein thrombosis (DVT), is the third most prevalent cardiovascular disorder and represents a significant public health challenge worldwide (1). With a yearly incidence of 1–2 cases per 1,000 adults, the global burden of VTE has been on a steady rise in recent years (2). Through epidemiologic studies, many risk factors have been identified as being related to VTE, such as advanced age, inflammation, cancer, major surgery, and trauma (2). Additionally, VTE risk can be influenced by several lifestyle factors such as smoking and body mass index (BMI).

Basal metabolic rate (BMR) is the minimum energy the body needs to perform basic physiological functions including breathing, circulation, heart rate, cellular growth, and brain function when at rest. Major factors affecting an individual’s BMR include age, genetics, body weight, environmental temperature, and health status (3). So far, limited studies assessed the association between BMR and cardiovascular diseases. The thickening of myointimal layer was observed in high-BMR mice compared with their counterparts (4). On the other hand, in humans, the level of BMR was found to be correlated with cardiovascular diseases such as aortic aneurysm, atrial fibrillation, and heart failure (5–8). Moreover, it is known that there is a close link between BMR and proinflammatory status, a critical promoter for VTE (9). These lines of evidence raise the question of whether there is a relationship between BMR and VTE. However, the unethical nature of attempting to control an individual’s basal metabolic rate and the long interval between exposure and outcome make it difficult to conduct randomized controlled trials (RCTs).

Mendelian randomization (MR) is a research technique using genetic information to study causal associations between phenotypes. It provides a means for investigating causality in the absence of RCTs, while also addressing key limitations of observational studies such as small sample sizes, unmeasured confounding, and reverse causation (10). We aimed to perform a two-sample MR to estimate whether genetically predicted BMR was causally associated with VTE and its subtypes (PE and DVT of lower extremities). As far as we know, this is the inaugural MR investigation focused on this matter.

## Methods

### Study design and data source

**Figure 1:**
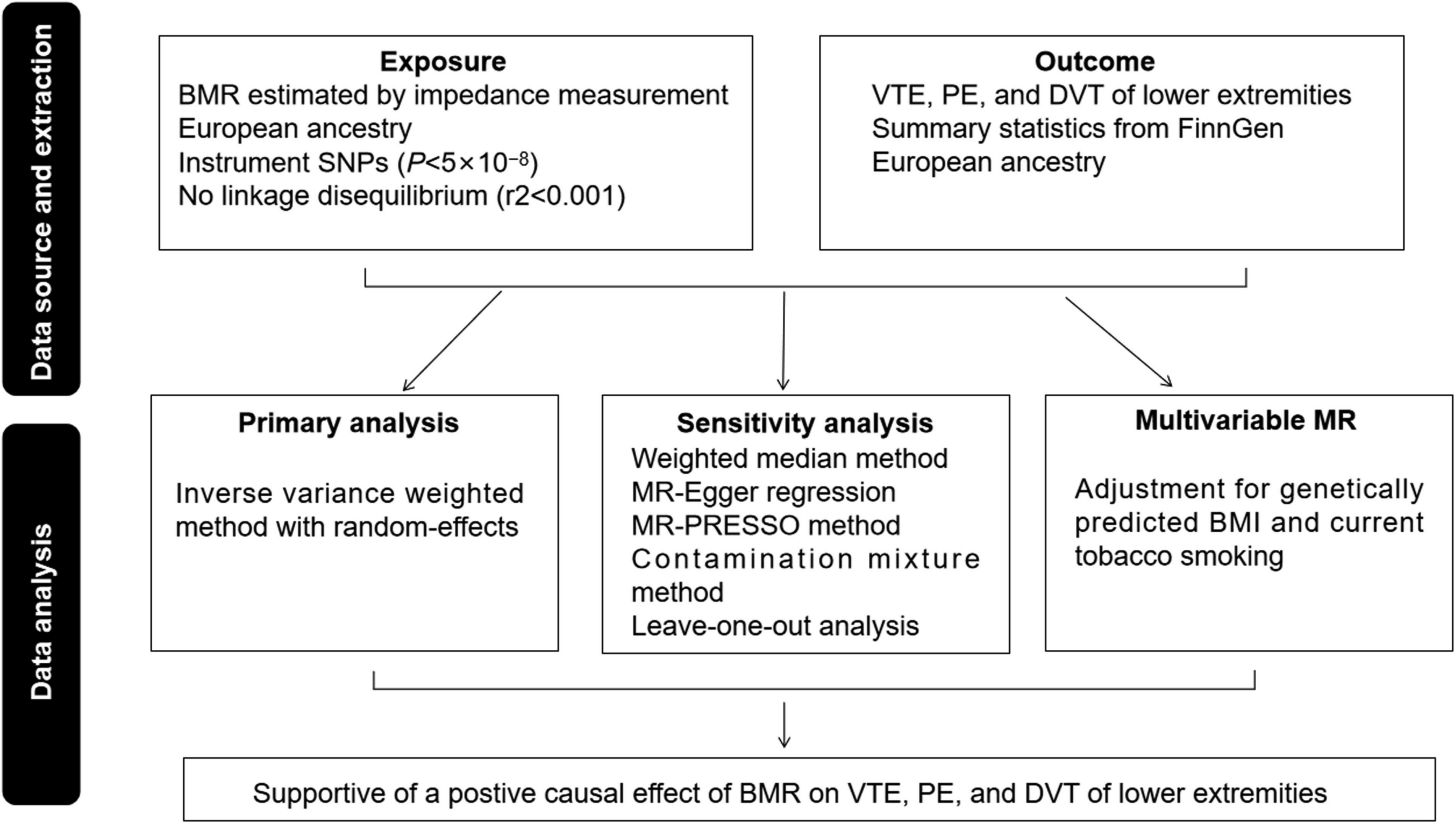
Overview of the study design.

Three fundamental assumptions underpinned the MR study: (1) the SNPs possess a close relationship with BMR, (2) the SNPs lack any association with confounders, and (3) the SNPs act on VTE solely via BMR. The research methodology is depicted in Figure 1. The STROBE-MR checklist was followed in reporting our methods and findings (Supplementary Table 1). Both the exposure and outcome summary statistics are available from the IEU GWAS database^1^ (11). Through a GWAS conducted by the University of Bristol’s MRC-IEU consortium (12), instrumental SNPs for BMR were obtained from up to 454,874 European individuals. BMR was estimated by impedance measurement at assessment centres of the UK Biobank. Kilojoules (kJ) were the units used to measure BMR. According to UK biobank data,^2^ the mean BMR level was 6597.38 kJ (SD 1357.83 kJ). Data concerning the associations of BMR-associated SNPs with VTE overall or its subtypes were generated by FinnGen (13). The cases and controls were restricted to those of European ancestry. Specifically, these datasets included 9,176 cases and 209,616 controls for VTE, 4,185 cases and 214,228 controls for PE, and 4,576 cases and 190,028 controls for DVT of lower extremities. The relevant information on the GWAS for BMR and VTE are summarized in Supplementary Table 2.

The original GWAS received ethical approval from their respective ethics committees, and prior to their involvement, each participant gave their written consent. Our analysis proceeded without the need for additional approval from the institutional review board, as only publicly available summary statistics were analyzed.

### Genetic instruments selection

Instrumental variables linked to BMR were extracted based on a rigorous significance threshold of 5 × 10^−8^. The TwoSampleMR package’s clump function was utilized to evaluate the extracted SNPs’ linkage disequilibrium (LD) (11), with data from the 1,000 Genomes LD reference panel comprising solely European participants. Using a 10,000 kb window and restricting pairwise LD r^2^ to <0.001 allowed us to ensure the independence of instrumental variables. Variant harmonization involved aligning betas from diverse GWAS examining the same effect allele. If it was unable to find a genetic variant within the outcome GWAS data, we chose proxy variants linked by LD with a minimum r^2^ value of 0.8 (14). Quantification of instrument strength involved the use of the F-statistic, while r-squared served to quantify the variance explained (15).

### Statistical analysis

The main approach was the inverse variance weighted method (multiplicative random-effect). This involved meta-analyzing the Wald estimates computed for each genetic variant’s effect, which were obtained as the ratio between the genetic variant’s associations with VTE and BMR. Under the premise of the inverse variance weighted method, it is expected that every instrument is deemed valid (16). The findings were presented as the odds ratio (OR) of VTE risk for a one standard deviation (SD) increase in genetically predicted BMR along with its respective 95% confidence interval (CI). Moreover, more robust techniques, comprising Mendelian randomization pleiotropy residual sum and outlier (MR-PRESSO), weighted median, MR-Egger, and contamination mixture method were utilized to overcome deviations from assumptions of MR arising owing to pleiotropy (17–21). The presence of pleiotropy can be inferred if the MR-Egger intercept value significantly deviates from zero. The existence of pleiotropy was also estimated applying MR-PRESSO which involves the exclusion of outlying SNPs from the instruments and re-assessment of effect estimates. Through the application of Cochran’s Q-statistic, the disparities in effects between instruments were examined. BMI and smoking are two important risk factors for VTE. To account for their possible effects modifying the causality between BMR and VTE, multivariable MR was performed as a supplementary analysis using the mv_multiple () function of the TwoSampleMR package. The meta-analysis conducted by Yengo and colleagues, which involved a total of 681,275 European individuals, yielded summary statistics pertaining to BMI (22). In regards to current tobacco smoking, we employed the results of a GWAS executed by the MRC-IEU consortium involving 462,434 European individuals (12). Supplementary Table 3 presents the relevant information on these datasets. Finally, the individual contributions of the instrumental SNPs to the IVW MR assessments were assessed using a leave-one-out analysis.

R software version 4.1.0 were employed to conduct all analyses, with the analysis involving the utilization of TwoSampleMR,^3^ MendelianRandomization,^4^ and MR-PRESSO^5^ packages (default settings). Taking into consideration multiple testing, a significance threshold of *p* < 0.017 (0.05/3) was established for the IVW after applying the Bonferroni correction. By following the methodology proposed by Brion et al. (23), the statistical power was determined.

## Results

### Overview of the instruments

Descriptive information on the instruments for BMR and their association with VTE and its subtypes is presented in Supplementary Tables 4–6. The particular features of these instrumental SNPs included the value of beta, standard error (SE), effect allele, effect allele frequency, other allele, *F*-statistic, etc. The *F*-statistics for each instrumental variable were observed to be higher than 16, surpassing the conventional limit of >10, which indicated satisfactory instrumental strength.

### Power analysis

With regards to the primary MR analysis, our study had 80% power to detect the smallest OR ranging from 1.160 to 1.234 for the effects of genetically predicted BMR on VTE and its subtypes (Supplementary Table 7).

### Primary MR analysis

**Figure 2:**
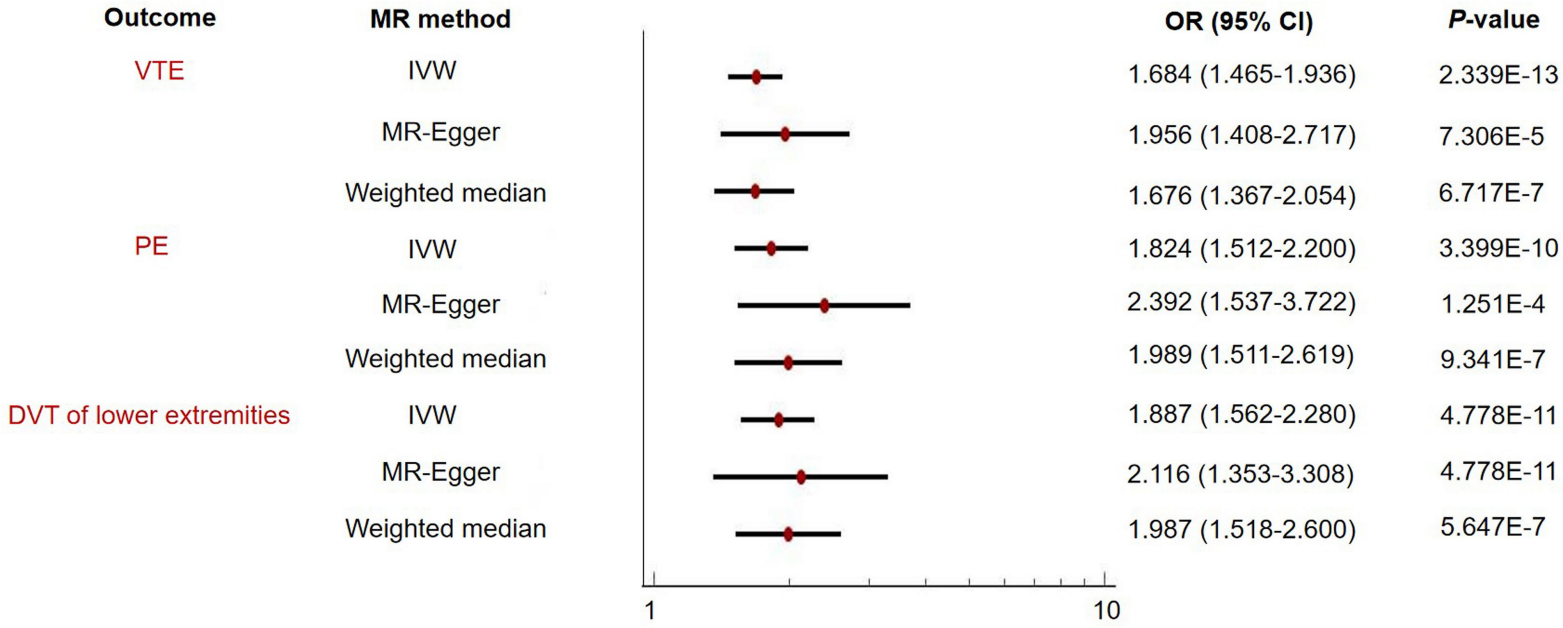
Two-sample Mendelian randomization (MR) analysis evaluating the relationship of basal metabolic rate with venous thromboembolism (VTE) and its subtypes including pulmonary embolism (PE) and deep vein thrombosis (DVT) of lower extremities.

The IVW MR analyses demonstrated that one standard deviation (SD) increase in genetically predicted BMR was associated with an increased risk of VTE (OR = 1.684, 95% CI: 1.465–1.936, *p* = 2.339 × 10^−13^), PE (OR = 1.824, 95% CI: 1.512–2.200, *p* = 3.399 × 10^−10^), and DVT of lower extremities (OR = 1.887, 95% CI: 1.562–2.280, *p* = 4.778 × 10^−11^) (Figure 2 and Table 1). A leave-one-out analysis using the IVW method found that no individual instrumental SNPs significantly influenced the MR estimates (Supplementary Tables 8–10).

**Table 1:** MR analysis evaluating the relationship between BMR and VTE.

Outcome	MR method	Number of instruments	OR	95% CI	*p*
Venous thromboembolism	IVW	498	1.684	1.465–1.936	2.339 × 10^−13^
	MR-Egger	498	1.956	1.408–2.717	7.306 × 10^−5^
	Weighted median	498	1.676	1.367–2.054	6.777 × 10^−7^
	MR-PRESSO (corrected)	497	1.669	1.455–1.914	1.656 × 10^−12^
Pulmonary embolism	IVW	498	1.824	1.512–2.200	3.399 × 10^−10^
	MR-Egger	498	2.392	1.537–3.722	1.251 × 10^−4^
	Weighted median	498	1.989	1.511–2.619	9.341 × 10^−7^
	MR-PRESSO (corrected)	496	1.822	1.516–2.190	3.524 × 10^−10^
DVT of lower extremities	IVW	498	1.887	1.562–2.280	4.778 × 10^−11^
	MR-Egger	498	2.116	1.353–3.308	1.086 × 10^−3^
	Weighted median	498	1.987	1.518–2.600	5.647 × 10^−7^
	MR-PRESSO (corrected)	496	1.827	1.515–2.204	6.789 × 10^−10^

MR, Mendelian randomization; CI, confidence interval; OR, odds ratio; VTE, venous thromboembolism; DVT, deep vein thrombosis; IVW, inverse variance weighted; BMR, basal metabolic rate.

### Sensitivity analysis and estimates for heterogeneity and pleiotropy

The MR-Egger and weighted median sensitivity analyses validated the IVW analyses, confirming the consistency of the results (Figure 2 and Table 1). We then proceeded to use MR-PRESSO to identify and exclude any possible instrumental variable outliers. Our MR-PRESSO outlier corrected method found genetically predicted BMR causally associated with VTE (OR = 1.669, 95% CI: 1.455–1.914, *p* = 1.656 × 10^−12^, removal of one SNP), PE (OR = 1.822, 95% CI: 1.516–2.190, *p* = 3.524 × 10^−10^, removal of two SNPs), and DVT of lower extremities (OR = 1.827, 95% CI: 1.515–2.204, *p* = 6.789 × 10^−10^, removal of two SNPs) (Figure 2 and Table 1), further supporting the IVW analyses. Additionally, the contamination mixture method was utilized for sensitivity analysis, allowing us to conduct MR efficiently and robustly even in the presence of invalid instrumental SNPs (20). The results also suggested that BMR was causally associated with VTE (OR = 1.572, 95% CI: 1.294–1.908, *p* = 1.53 × 10^−5^), PE (OR = 1.706, 95% CI: 1.378–2.095, *p* = 1.59 × 10^−5^), and DVT of lower extremities (OR = 2.132, 95% CI: 1.679–2.708, *p* = 3.08 × 10^−8^). Heterogeneity was observed (*p*-value<0.001) in our analysis; however, the MR-Egger intercept did not reveal any signs of directional pleiotropy (Table 2).

**Table 2:** Pleiotropy estimation using MR-Egger intercept.

Outcome	MR-Egger		
	Intercept	SE	*p*-value
Venous thromboembolism	−0.002	0.002	0.329
Pulmonary embolism	−0.004	0.003	0.185
DVT of lower extremities	−0.002	0.003	0.580

SE, standard error; DVT, deep vein thrombosis; MR, Mendelian randomization.

BMI and smoking are recognized as significant contributing factors to VTE. To account for the influence of BMI and current tobacco smoking, we employed multivariable MR analysis in our study. Multivariable MR analysis demonstrated independent positive effects of BMR on VTE, PE, and DVT of lower extremities (Table 3).

**Table 3:** Multivarible MR analysis for estimating the causal effect of BMR on VTE and its subtypes.

Outcome	Adjustment for	Number of SNPs for BMR included in multivarible MR	OR	95% CI	*p*-value
VTE	BMI and current tobacco smoking	291	1.516	1.206–1.903	7.873 × 10^−4^
PE	BMI and current tobacco smoking	291	1.754	1.291–2.384	6.060 × 10^−4^
DVT of lower extremities	BMI and current tobacco smoking	291	1.781	1.308–2.423	8.021 × 10^−4^

BMI, body mass index; BMR, basal metabolic rate; CI, confidence interval; DVT, deep vein thrombosis; MR, Mendelian randomization; OR, odds ratio; PE, pulmonary embolism; VTE, venous thromboembolism.

### Reverse MR

Finally, we evaluated the causal effects of VTE and its subtypes on BMR. The number of instrument SNPs predicting VTE, PE and DVT of lower extremities were 13, 6, and 9, respectively. Supplementary Table 11 shows the characteristics of these instrument SNPs. Our MR results did not uncover any indications of reverse causation (Supplementary Table 12). No evidence of pleiotropy was revealed using the MR-Egger intercepts (Supplementary Table 12).

## Discussion

Utilizing GWAS summary-level data, we conducted a MR analysis to investigate the causal relationship between genetically predicted BMR and venous thromboembolism (VTE), including its subtypes. Our results were supportive of BMR having positive effects on VTE, PE, and DVT of lower extremities. A series of sensitivity analyses reinforced the validity of the results. As far as we know, the current analysis represents the inaugural exploration of the causal relationship between BMR and VTE, providing novel insights into the predisposition to VTE.

Our findings coincided with the outcomes of prior animal studies, observational investigations and an MR research, all of which indicated a significant relationship between BMR and cardiovascular diseases (4–8, 24). In their research using a mouse model, Sawicka et al. observed that mice with a high BMR displayed a notably thicker middle layer of the aorta in comparison to those with a low BMR; this change contributed to arterial stiffness and the development of cardiovascular diseases (4). In addition, some research groups revealed an association between BMR and human cardiovascular diseases, including heart failure, aortic aneurysm, and atrial fibrillation, through a cross-sectional study design (5–8). A recent MR investigation performed by Li and colleagues reported a causal link between BMR and common cardiovascular diseases including atrial fibrillation and flutter, myocardial infarction, heart failure, and aortic aneurysm (24). Our research provided important supplementary evidence to the existing relationship between BMR and cardiovascular diseases. It confirmed that in addition to the above-mentioned cardiovascular diseases, BMR was also a risk factor for VTE and its subtypes.

An elevated risk of VTE may be attributed to some potential mechanisms associated with an increased BMR. Firstly, reactive oxygen species (ROS) are produced as a detrimental byproduct during cellular metabolism. Elevated BMR can be indicative of a high intrinsic metabolic activity, potentially resulting in an augmented production of ROS. Excessive ROS production can directly impair endothelial cell function by disrupting normal endothelial cell signaling and gene expression and damaging cellular components, such as lipids, proteins, and DNA (25). ROS can also stimulate the formation of oxidative stress, enhance the production and secretion of vasoconstrictor molecules, and promote inflammation within the endothelium (25). These processes ultimately result in endothelial dysfunction, thereby promoting a pro-inflammatory and pro-coagulant milieu in the vasculature, consequently heightening the predisposition to VTE. Secondly, high BMR may promote platelet activation and aggregation which are important in determining a prothrombotic state. Studies have reported an association between a high metabolic rate and changes in different hormones and metabolites levels in the body, such as higher levels of adrenaline and catecholamines (26, 27). These substances can enhance platelet function and promote aggregation. The increased metabolic rate may also induce alterations in blood flow and shear stress within the vasculature (28, 29), which can activate platelets and facilitate their aggregation at sites of vascular injury or dysfunction. In addition, high BMR-induced excessive ROS production can play a crucial role in platelet activation and aggregation. Third, among individuals of normal weight and those who are overweight, there is a positive link between BMR and proinflammatory status (30, 31). Elevated BMR can contribute to the generation of inflammatory cytokines such as interleukin (IL)-6 and interferon (IFN)-γ through inducing ROS production (32). Moreover, alterations in metabolic pathways and hormonal imbalances associated with high BMR, such as insulin resistance and dyslipidemia, may also contribute to a proinflammatory state (33–35). It is known that a proinflammatory state plays an important role in VTE development (36). Further investigation is necessary to fully understand the underlying pathways by which an elevated BMR influences the susceptibility to VTE.

The implications of this study could extend to clinical practice, particularly in the context of preventing and managing VTE. Current strategies for the prevention of VTE include pharmacological prophylaxis, mechanical prophylaxis, and early mobilization, among others. However, these strategies may not be effective for all patients and may also be associated with adverse effects. For example, the use of anticoagulant medications, such as heparin or low molecular weight heparin, can raise the bleeding probability (37). Our MR findings implied that reducing BMR might be a promising strategy for preventing VTE. This could be achieved through lifestyle modifications, such as weight management and caloric restriction. Future studies could investigate the interactions of these interventions with BMR in reducing the risk of VTE.

There are several limitations to this study. Firstly, horizontal pleiotropy poses a major constraint in MR design, as it indicates that instrumental variables employed for MR investigations have an impact on outcomes through alternative pathways instead of the intended exposure. However, it is unlikely that pleiotropic effects would significantly impact the validity of this study, as the sensitivity analysis using alternative MR methods produced congruent results. Additionally, the MR-Egger intercept did not reveal any evidence of horizontal pleiotropy, and the multivariable MR identified notable relationships. Secondly, it should be noted that our MR study only evaluated the long-term impact of BMR on VTE and its subtypes. This is because MR serves as a statistical method to investigate how long-term differences in exposures can influence the risk of developing diseases. We did not evaluate the short-term contribution of BMR to VTE and its subtypes. Thirdly, in our study, the assessment of the association between BMR and VTE, including its subtypes, was limited to the linear relationship and relied exclusively on GWAS summary statistics. The lack of access to individual-level data prohibited us from evaluating a nonlinear association. Fourthly, we focused on the association of BMR with VTE, PE, and DVT of lower extremities; other subtypes of VTE, such as cerebral venous thrombosis, were not included in our analysis. Fifthly, considering that our analyses solely included individuals of European descent, it is important to interpret these findings with caution regarding their applicability to other populations. Additional research is required to evaluate this aspect.

In conclusion, our MR study has provided the first-ever evidence that genetically predicted BMR has a positive causal impact on VTE and its subtypes, including PE and DVT of lower extremities. Modulating BMR might hold promise as a clinically relevant intervention to prevent VTE and its subtypes.

## Data availability statement

The IEU Open GWAS Project database (https://gwas.mrcieu.ac.uk/) provides public access to all datasets analyzed in the present study. Further inquiries can be directed to the corresponding author.

## Author contributions

JH: Conceptualization, Data curation, Formal analysis, Investigation, Methodology, Project administration, Resources, Software, Validation, Visualization, Writing – original draft, Writing – review & editing. YX: Funding acquisition, Supervision, Writing – review & editing.

## Footnotes

^1^ https://gwas.mrcieu.ac.uk/

^2^ https://biobank.ctsu.ox.ac.uk/crystal/field.cgi?id=23105

^3^ https://mrcieu.github.io/TwoSampleMR/index.html

^4^ https://github.com/cran/MendelianRandomization

^5^ https://github.com/rondolab/MR-PRESSO
