# Supplementary material for: Genetically predicted basal metabolic rate and venous thromboembolism risk: a Mendelian randomization study
Source: Front Nutr. 2023 Dec 21;10:1263804. doi: 10.3389/fnut.2023.1263804 (PMC10768029; doi:10.3389/fnut.2023.1263804)
Supplement: Supplementary file 2 [file Table_2.DOCX]

Supplementary Table 2 Characteristics of the GWAS datasets for exposure and outcome.

| Trait | ID for GWAS dataset | Author or Consortium | Ethnicity | Sample size | Number of SNPs |
| --- | --- | --- | --- | --- | --- |
| Basal metabolic rate | ukb-b-16446 | MRC-IEU | European | 454,874 individuals | 9,851,867 |
| Venous thromboembolism | finn-b-I9_VTE | FinnGen | European | 9,176 cases and 209,616 controls | 16,380,466 |
| Pulmonary embolism | finn-b-I9_PULMEMB | FinnGen | European | 4,185 cases and 214,228 controls | 16,380,466 |
| DVT of lower extremities | finn-b-I9_PHLETHROMBDVTLOW | FinnGen | European | 4,576 cases and 190,028 controls | 16,380,409 |

IV, instrumental variable; IVW, inverse variance weighted; DVT, deep vein thrombosis; GWAS, genome-wide association study; MR, Mendelian randomization; MRC-IEU, Medical Research Council-Integrative Epidemiology Unit; SNP, single nucleotide polymorphism; VTE, venous thromboembolism.
