# Supplementary material for: Genetically predicted basal metabolic rate and venous thromboembolism risk: a Mendelian randomization study
Source: Front Nutr. 2023 Dec 21;10:1263804. doi: 10.3389/fnut.2023.1263804 (PMC10768029; doi:10.3389/fnut.2023.1263804)
Supplement: Supplementary file 3 [file Table_3.DOCX]

Supplementary Table 3 Characteristics of the GWAS datasets for BMI and current tobacco smoking.

| Trait | ID for GWAS dataset | Author or Consortium | Ethnicity | Sample size | Number of SNPs |
| --- | --- | --- | --- | --- | --- |
| BMI | ieu-b-40 | GIANT | European | 681,275 individuals | 2,336,260 |
| Current tobacco smoking | ukb-b-223 | MRC-IEU | European | 462,434 individuals | 9,851,867 |

BMI, body mass index; IV, instrumental variables; MR, Mendelian randomization; SNP, single nucleotide polymorphism; GWAS, genome-wide association study; MRC-IEU, Medical Research Council-Integrative Epidemiology Unit; GIANT, Genetic Investigation of ANthropometric Traits.
