# Supplementary material for: Genetically predicted basal metabolic rate and venous thromboembolism risk: a Mendelian randomization study
Source: Front Nutr. 2023 Dec 21;10:1263804. doi: 10.3389/fnut.2023.1263804 (PMC10768029; doi:10.3389/fnut.2023.1263804)
Supplement: Supplementary file 5 [file Table_5.DOCX]

Supplementary Table 5 Characteristics of the instrumental variables for BMR and their relationship with PE.

| Position | Chr | SNP | Effect allele | Other allele | EAF | Exposure effect |  |  | F-statistic | Outcome effect |  |  |
| --- | --- | --- | --- | --- | --- | --- | --- | --- | --- | --- | --- | --- |
|  |  |  |  |  |  | Effect size (β) | SE | *P*-value |  | Effect size (β) | SE | *P*-value |
| 69353863 | 4 | rs10020631 | A | G | 0.248 | -0.008 | 0.002 | 3.10E-08 | 16 | -0.026 | 0.027 | 0.329 |
| 277617 | 5 | rs10058393 | T | C | 0.129 | 0.013 | 0.002 | 5.90E-11 | 42 | 0.001 | 0.039 | 0.974 |
| 113828811 | 9 | rs1008158 | G | A | 0.342 | 0.009 | 0.001 | 7.30E-10 | 81 | 0.032 | 0.023 | 0.165 |
| 36780549 | 2 | rs10172196 | A | G | 0.306 | 0.012 | 0.001 | 8.80E-18 | 144 | 0.021 | 0.024 | 0.382 |
| 58885575 | 2 | rs10176567 | T | C | 0.143 | 0.011 | 0.002 | 8.00E-09 | 30 | -0.031 | 0.034 | 0.358 |
| 142297493 | 2 | rs10188231 | G | C | 0.186 | -0.011 | 0.002 | 1.50E-11 | 30 | -0.014 | 0.028 | 0.603 |
| 42575820 | 2 | rs10202845 | G | A | 0.113 | -0.017 | 0.002 | 1.50E-16 | 72 | 0.027 | 0.037 | 0.458 |
| 150668070 | 7 | rs10236214 | T | C | 0.642 | 0.019 | 0.001 | 1.00E-45 | 361 | -0.027 | 0.025 | 0.268 |
| 121963813 | 7 | rs10248298 | A | C | 0.366 | 0.012 | 0.001 | 3.10E-20 | 144 | 0.036 | 0.023 | 0.122 |
| 55884295 | 7 | rs10260993 | G | T | 0.196 | -0.010 | 0.002 | 7.60E-09 | 25 | -0.018 | 0.026 | 0.491 |
| 92253972 | 7 | rs10269774 | A | G | 0.326 | 0.026 | 0.001 | 1.60E-77 | 676 | 0.067 | 0.025 | 0.007 |
| 3445721 | 19 | rs10401784 | A | C | 0.640 | -0.011 | 0.001 | 1.10E-14 | 121 | -0.063 | 0.023 | 0.007 |
| 18834514 | 19 | rs10404726 | T | C | 0.466 | -0.009 | 0.001 | 2.90E-11 | 81 | -0.034 | 0.023 | 0.135 |
| 4015316 | 16 | rs1045475 | G | A | 0.821 | 0.020 | 0.002 | 9.90E-33 | 100 | -0.010 | 0.029 | 0.731 |
| 126083658 | 6 | rs10457469 | A | G | 0.523 | 0.015 | 0.001 | 9.30E-29 | 225 | 0.006 | 0.023 | 0.790 |
| 211540507 | 2 | rs1047891 | A | C | 0.316 | 0.017 | 0.001 | 1.30E-33 | 289 | 0.053 | 0.024 | 0.029 |
| 80599757 | 3 | rs10511111 | C | T | 0.309 | 0.009 | 0.001 | 2.30E-10 | 81 | -0.002 | 0.025 | 0.939 |
| 198950240 | 2 | rs1064213 | A | G | 0.478 | 0.011 | 0.001 | 2.80E-18 | 121 | 0.076 | 0.023 | 0.001 |
| 22058137 | 10 | rs10740991 | C | G | 0.718 | -0.010 | 0.001 | 1.80E-11 | 100 | -0.035 | 0.026 | 0.167 |
| 69827658 | 12 | rs10748128 | T | G | 0.345 | 0.012 | 0.001 | 1.20E-17 | 144 | 0.013 | 0.024 | 0.579 |
| 122939655 | 12 | rs10773172 | A | G | 0.742 | 0.019 | 0.001 | 2.80E-38 | 361 | -0.018 | 0.029 | 0.537 |
| 88806348 | 16 | rs10775348 | G | A | 0.704 | 0.013 | 0.001 | 5.70E-19 | 169 | 0.020 | 0.025 | 0.415 |
| 46197755 | 17 | rs10775406 | G | A | 0.760 | -0.010 | 0.002 | 5.80E-11 | 25 | -0.008 | 0.026 | 0.752 |
| 97792792 | 12 | rs10777860 | A | G | 0.528 | -0.011 | 0.001 | 6.00E-17 | 121 | -0.006 | 0.023 | 0.801 |
| 183228114 | 2 | rs10803955 | G | A | 0.508 | -0.011 | 0.001 | 1.70E-16 | 121 | -0.025 | 0.023 | 0.282 |
| 66697966 | 10 | rs10822373 | T | C | 0.504 | -0.007 | 0.001 | 3.20E-08 | 49 | 0.034 | 0.023 | 0.131 |
| 29529523 | 12 | rs10843397 | T | C | 0.242 | 0.009 | 0.002 | 1.40E-09 | 20 | 0.002 | 0.027 | 0.930 |
| 114999636 | 13 | rs10870597 | G | A | 0.235 | -0.011 | 0.002 | 8.50E-13 | 30 | 0.047 | 0.027 | 0.075 |
| 121175524 | 10 | rs10886477 | A | G | 0.128 | 0.011 | 0.002 | 1.00E-08 | 30 | -0.041 | 0.044 | 0.348 |
| 88030441 | 10 | rs10887571 | T | C | 0.448 | 0.008 | 0.001 | 1.60E-09 | 64 | 0.040 | 0.023 | 0.078 |
| 32125943 | 1 | rs10914462 | G | A | 0.427 | -0.010 | 0.001 | 2.40E-15 | 100 | -0.009 | 0.023 | 0.691 |
| 45182527 | 4 | rs10938397 | G | A | 0.434 | 0.014 | 0.001 | 3.50E-28 | 196 | 0.014 | 0.023 | 0.531 |
| 18483405 | 4 | rs10939792 | G | C | 0.320 | 0.009 | 0.001 | 1.50E-10 | 81 | 0.000 | 0.026 | 0.998 |
| 111687379 | 9 | rs10979612 | C | T | 0.073 | 0.018 | 0.003 | 3.90E-13 | 36 | -0.062 | 0.036 | 0.083 |
| 52772113 | 10 | rs10995366 | A | G | 0.251 | -0.010 | 0.002 | 1.50E-10 | 25 | 0.045 | 0.026 | 0.086 |
| 25178864 | 10 | rs11014285 | A | G | 0.165 | 0.018 | 0.002 | 7.30E-25 | 81 | -0.027 | 0.038 | 0.480 |
| 129248044 | 10 | rs11018104 | A | T | 0.409 | 0.007 | 0.001 | 3.60E-08 | 49 | 0.015 | 0.024 | 0.545 |
| 27705188 | 11 | rs11030112 | A | G | 0.319 | 0.019 | 0.001 | 3.30E-42 | 361 | 0.035 | 0.024 | 0.152 |
| 28665359 | 12 | rs11049684 | T | C | 0.299 | -0.011 | 0.001 | 5.10E-14 | 121 | -0.004 | 0.026 | 0.891 |
| 120520863 | 12 | rs11065015 | T | C | 0.027 | -0.032 | 0.004 | 1.10E-14 | 64 | 0.040 | 0.052 | 0.445 |
| 112059557 | 12 | rs11065979 | T | C | 0.438 | -0.014 | 0.001 | 3.30E-25 | 196 | 0.017 | 0.023 | 0.479 |
| 218634787 | 1 | rs1108548 | G | A | 0.277 | 0.015 | 0.001 | 2.60E-25 | 225 | 0.002 | 0.025 | 0.931 |
| 102709308 | 4 | rs11097755 | C | T | 0.443 | 0.009 | 0.001 | 3.50E-12 | 81 | -0.011 | 0.023 | 0.639 |
| 170865229 | 5 | rs111365325 | T | C | 0.231 | -0.014 | 0.002 | 1.10E-20 | 49 | -0.086 | 0.028 | 0.002 |
| 73757155 | 9 | rs11142700 | C | T | 0.408 | -0.008 | 0.001 | 2.10E-10 | 64 | 0.022 | 0.024 | 0.366 |
| 109653825 | 6 | rs11153171 | T | C | 0.356 | -0.013 | 0.001 | 1.40E-21 | 169 | 0.005 | 0.023 | 0.846 |
| 171635471 | 4 | rs111598585 | T | C | 0.209 | -0.009 | 0.002 | 9.60E-09 | 20 | -0.083 | 0.037 | 0.025 |
| 101186641 | 14 | rs11160601 | T | C | 0.092 | 0.017 | 0.002 | 9.90E-14 | 72 | -0.004 | 0.032 | 0.897 |
| 30290357 | 19 | rs111640872 | C | G | 0.331 | 0.016 | 0.001 | 7.10E-29 | 256 | 0.044 | 0.024 | 0.068 |
| 66118509 | 12 | rs11175890 | T | C | 0.267 | -0.009 | 0.002 | 5.70E-09 | 20 | 0.011 | 0.031 | 0.714 |
| 115949262 | 10 | rs11196652 | A | G | 0.244 | 0.010 | 0.002 | 4.60E-10 | 25 | -0.020 | 0.028 | 0.466 |
| 150249101 | 1 | rs11205354 | A | C | 0.444 | -0.008 | 0.001 | 3.10E-09 | 64 | -0.010 | 0.023 | 0.660 |
| 1034997 | 4 | rs112069922 | T | C | 0.048 | -0.026 | 0.003 | 4.70E-17 | 75 | -0.020 | 0.068 | 0.765 |
| 120239937 | 11 | rs11217843 | G | A | 0.163 | -0.013 | 0.002 | 2.30E-13 | 42 | -0.084 | 0.027 | 0.002 |
| 7719065 | 6 | rs11243202 | C | T | 0.486 | 0.018 | 0.001 | 6.30E-42 | 324 | 0.004 | 0.023 | 0.847 |
| 126658075 | 10 | rs11245450 | A | G | 0.422 | -0.011 | 0.001 | 6.80E-17 | 121 | -0.026 | 0.023 | 0.259 |
| 20222686 | 2 | rs112544217 | T | C | 0.022 | -0.026 | 0.005 | 5.40E-09 | 27 | 0.051 | 0.094 | 0.586 |
| 93112924 | 14 | rs112560164 | A | G | 0.191 | 0.011 | 0.002 | 3.70E-11 | 30 | 0.018 | 0.033 | 0.586 |
| 155822629 | 1 | rs112685832 | A | C | 0.115 | 0.013 | 0.002 | 7.30E-11 | 42 | -0.046 | 0.040 | 0.242 |
| 14296329 | 11 | rs112758380 | A | G | 0.057 | 0.018 | 0.003 | 1.30E-10 | 36 | 0.041 | 0.035 | 0.242 |
| 126506694 | 8 | rs112875651 | A | G | 0.391 | 0.012 | 0.001 | 2.10E-19 | 144 | -0.004 | 0.023 | 0.867 |
| 36220876 | 14 | rs112957890 | G | A | 0.265 | 0.010 | 0.001 | 5.40E-12 | 100 | -0.001 | 0.027 | 0.980 |
| 46399175 | 22 | rs113619763 | T | A | 0.062 | 0.017 | 0.003 | 1.10E-09 | 32 | 0.012 | 0.050 | 0.816 |
| 41877671 | 6 | rs114056237 | A | G | 0.012 | -0.061 | 0.006 | 1.90E-25 | 103 | 0.101 | 0.182 | 0.580 |
| 78555512 | 17 | rs114177791 | T | G | 0.214 | 0.011 | 0.002 | 1.20E-12 | 30 | -0.010 | 0.029 | 0.731 |
| 33348679 | 2 | rs115179432 | G | A | 0.072 | -0.023 | 0.003 | 6.20E-19 | 59 | -0.028 | 0.049 | 0.572 |
| 138817193 | 7 | rs11525873 | C | T | 0.098 | -0.014 | 0.002 | 6.30E-11 | 49 | 0.022 | 0.032 | 0.502 |
| 70315987 | 2 | rs11545482 | T | C | 0.020 | -0.031 | 0.005 | 8.70E-12 | 38 | 0.069 | 0.158 | 0.662 |
| 23425139 | 1 | rs11578046 | A | G | 0.327 | -0.013 | 0.001 | 2.40E-21 | 169 | -0.024 | 0.024 | 0.324 |
| 150497496 | 7 | rs115946508 | A | C | 0.111 | -0.013 | 0.002 | 2.70E-10 | 42 | -0.029 | 0.042 | 0.501 |
| 133380790 | 12 | rs11611651 | A | G | 0.088 | 0.017 | 0.002 | 9.70E-14 | 72 | -0.013 | 0.054 | 0.815 |
| 21493853 | 13 | rs11616283 | C | T | 0.140 | 0.012 | 0.002 | 3.60E-10 | 36 | 0.019 | 0.037 | 0.611 |
| 62610556 | 20 | rs116165844 | T | G | 0.137 | -0.011 | 0.002 | 9.30E-09 | 30 | -0.002 | 0.036 | 0.963 |
| 183954625 | 2 | rs116337081 | T | C | 0.070 | 0.014 | 0.003 | 3.70E-08 | 22 | 0.004 | 0.054 | 0.939 |
| 32762592 | 18 | rs11663903 | A | G | 0.429 | 0.008 | 0.001 | 6.40E-09 | 64 | 0.022 | 0.023 | 0.344 |
| 219835489 | 2 | rs11684531 | G | A | 0.133 | -0.011 | 0.002 | 1.60E-08 | 30 | 0.001 | 0.044 | 0.977 |
| 241843564 | 2 | rs11688707 | A | G | 0.364 | 0.008 | 0.001 | 1.30E-09 | 64 | -0.021 | 0.023 | 0.361 |
| 53531085 | 14 | rs117034105 | C | T | 0.049 | 0.018 | 0.003 | 2.60E-08 | 36 | 0.015 | 0.051 | 0.772 |
| 131551027 | 3 | rs11709402 | G | A | 0.279 | 0.012 | 0.001 | 1.10E-15 | 144 | 0.019 | 0.026 | 0.466 |
| 32723478 | 5 | rs11739036 | A | G | 0.343 | -0.012 | 0.001 | 4.50E-17 | 144 | -0.031 | 0.024 | 0.195 |
| 17245591 | 12 | rs117451679 | G | A | 0.106 | 0.014 | 0.002 | 2.80E-10 | 49 | -0.021 | 0.035 | 0.539 |
| 79543740 | 10 | rs117543413 | T | C | 0.018 | -0.038 | 0.005 | 2.40E-14 | 58 | -0.025 | 0.094 | 0.789 |
| 120067932 | 6 | rs11756675 | G | A | 0.257 | 0.008 | 0.001 | 1.50E-08 | 64 | -0.054 | 0.029 | 0.063 |
| 81221014 | 13 | rs1176314 | G | T | 0.550 | -0.008 | 0.001 | 5.10E-09 | 64 | -0.009 | 0.023 | 0.712 |
| 10801857 | 8 | rs11777007 | T | C | 0.547 | -0.011 | 0.001 | 1.10E-16 | 121 | -0.075 | 0.035 | 0.029 |
| 23391493 | 8 | rs11785562 | A | G | 0.201 | 0.012 | 0.002 | 5.60E-14 | 36 | 0.028 | 0.026 | 0.282 |
| 23345347 | 9 | rs11794152 | G | A | 0.415 | 0.011 | 0.001 | 4.20E-16 | 121 | 0.029 | 0.023 | 0.210 |
| 90322922 | 6 | rs1179905 | G | A | 0.195 | 0.011 | 0.002 | 2.40E-11 | 30 | 0.014 | 0.030 | 0.631 |
| 28356600 | 22 | rs118173451 | C | T | 0.016 | -0.031 | 0.005 | 6.80E-09 | 38 | 0.076 | 0.094 | 0.418 |
| 2176403 | 19 | rs11880992 | A | G | 0.408 | 0.013 | 0.001 | 4.10E-24 | 169 | 0.023 | 0.024 | 0.319 |
| 49401982 | 8 | rs11997525 | A | T | 0.167 | 0.015 | 0.002 | 2.70E-17 | 56 | -0.023 | 0.033 | 0.480 |
| 49364360 | 1 | rs12047401 | G | T | 0.679 | -0.008 | 0.001 | 2.30E-08 | 64 | 0.078 | 0.024 | 0.001 |
| 11252716 | 1 | rs1205593 | C | T | 0.759 | -0.016 | 0.002 | 4.10E-25 | 64 | -0.049 | 0.027 | 0.068 |
| 118881689 | 1 | rs12083887 | G | A | 0.598 | 0.007 | 0.001 | 4.30E-08 | 49 | 0.030 | 0.023 | 0.191 |
| 203791049 | 1 | rs12123505 | G | C | 0.719 | -0.009 | 0.001 | 2.20E-09 | 81 | 0.044 | 0.024 | 0.071 |
| 120191044 | 1 | rs12129705 | T | A | 0.127 | -0.012 | 0.002 | 6.30E-09 | 36 | -0.052 | 0.035 | 0.141 |
| 62579891 | 1 | rs12140153 | T | G | 0.094 | -0.020 | 0.002 | 8.70E-18 | 100 | -0.057 | 0.042 | 0.180 |
| 144848756 | 8 | rs12156265 | A | G | 0.590 | 0.008 | 0.001 | 3.20E-10 | 64 | -0.018 | 0.023 | 0.441 |
| 60720682 | 5 | rs12188627 | G | A | 0.488 | -0.011 | 0.001 | 7.20E-16 | 121 | 0.009 | 0.023 | 0.697 |
| 83487443 | 6 | rs12200061 | A | G | 0.405 | -0.008 | 0.001 | 4.40E-09 | 64 | 0.026 | 0.024 | 0.275 |
| 76164589 | 6 | rs12209223 | A | C | 0.101 | 0.015 | 0.002 | 1.70E-12 | 56 | -0.002 | 0.033 | 0.952 |
| 12131542 | 6 | rs12213070 | A | G | 0.346 | -0.009 | 0.001 | 6.00E-12 | 81 | -0.065 | 0.024 | 0.007 |
| 19028623 | 6 | rs12216497 | T | C | 0.561 | -0.011 | 0.001 | 9.00E-18 | 121 | -0.026 | 0.024 | 0.275 |
| 47951353 | 11 | rs1228024 | A | C | 0.661 | -0.010 | 0.001 | 1.50E-13 | 100 | -0.041 | 0.025 | 0.100 |
| 12890358 | 11 | rs12294689 | C | A | 0.104 | 0.014 | 0.002 | 2.80E-10 | 49 | 0.083 | 0.061 | 0.173 |
| 134601012 | 11 | rs12364470 | G | T | 0.165 | 0.011 | 0.002 | 9.10E-11 | 30 | 0.031 | 0.027 | 0.261 |
| 90213070 | 12 | rs12427047 | T | C | 0.243 | -0.011 | 0.002 | 8.00E-14 | 30 | 0.047 | 0.024 | 0.052 |
| 37234008 | 9 | rs12553221 | A | G | 0.362 | 0.008 | 0.001 | 1.60E-08 | 64 | -0.032 | 0.024 | 0.176 |
| 27730940 | 2 | rs1260326 | C | T | 0.604 | 0.018 | 0.001 | 2.50E-42 | 324 | -0.016 | 0.024 | 0.515 |
| 36787962 | 5 | rs12657771 | A | G | 0.439 | -0.013 | 0.001 | 4.80E-23 | 169 | -0.019 | 0.023 | 0.413 |
| 207029825 | 2 | rs12694042 | T | C | 0.502 | -0.008 | 0.001 | 3.90E-10 | 64 | 0.010 | 0.023 | 0.682 |
| 23896049 | 2 | rs12713004 | G | A | 0.725 | 0.016 | 0.001 | 3.70E-27 | 256 | 0.003 | 0.030 | 0.926 |
| 202013757 | 1 | rs12731187 | T | C | 0.358 | -0.008 | 0.001 | 1.20E-09 | 64 | 0.023 | 0.023 | 0.319 |
| 97067652 | 1 | rs12731454 | G | A | 0.309 | 0.011 | 0.001 | 1.70E-15 | 121 | 0.039 | 0.026 | 0.133 |
| 104508202 | 10 | rs12763284 | G | A | 0.466 | 0.012 | 0.001 | 2.70E-21 | 144 | 0.035 | 0.023 | 0.125 |
| 5042294 | 10 | rs12764498 | C | T | 0.118 | -0.014 | 0.002 | 2.20E-12 | 49 | -0.094 | 0.034 | 0.005 |
| 123493123 | 12 | rs12820906 | G | A | 0.247 | 0.012 | 0.002 | 3.60E-15 | 36 | -0.049 | 0.028 | 0.082 |
| 25927832 | 14 | rs12879423 | G | A | 0.679 | 0.015 | 0.001 | 8.30E-28 | 225 | -0.019 | 0.024 | 0.417 |
| 103256877 | 14 | rs12888955 | A | G | 0.657 | -0.009 | 0.001 | 1.30E-10 | 81 | -0.005 | 0.023 | 0.835 |
| 42894143 | 14 | rs12889702 | C | A | 0.313 | 0.010 | 0.001 | 5.70E-12 | 100 | -0.039 | 0.025 | 0.114 |
| 86276000 | 15 | rs12907384 | C | T | 0.532 | -0.011 | 0.001 | 4.70E-17 | 121 | -0.012 | 0.023 | 0.604 |
| 62186447 | 15 | rs12908182 | T | C | 0.464 | -0.010 | 0.001 | 2.80E-15 | 100 | 0.005 | 0.024 | 0.831 |
| 406427 | 16 | rs12926311 | C | G | 0.354 | -0.009 | 0.001 | 2.80E-10 | 81 | 0.002 | 0.025 | 0.939 |
| 15139051 | 16 | rs12934835 | G | A | 0.309 | -0.013 | 0.001 | 5.60E-19 | 169 | -0.012 | 0.024 | 0.611 |
| 137083193 | 4 | rs1296328 | C | A | 0.559 | -0.010 | 0.001 | 2.30E-14 | 100 | 0.006 | 0.023 | 0.793 |
| 51091385 | 20 | rs13043303 | A | G | 0.183 | -0.020 | 0.002 | 1.00E-32 | 100 | 0.014 | 0.031 | 0.656 |
| 38601237 | 3 | rs13059004 | C | A | 0.478 | 0.009 | 0.001 | 4.60E-12 | 81 | 0.004 | 0.023 | 0.871 |
| 171129859 | 3 | rs13085472 | C | T | 0.666 | -0.009 | 0.001 | 5.70E-10 | 81 | -0.042 | 0.024 | 0.078 |
| 54351220 | 4 | rs13125807 | T | C | 0.142 | 0.012 | 0.002 | 4.20E-11 | 36 | 0.037 | 0.028 | 0.191 |
| 103457789 | 7 | rs13225455 | C | A | 0.226 | 0.009 | 0.002 | 4.00E-08 | 20 | 0.020 | 0.028 | 0.464 |
| 72973854 | 7 | rs13244614 | A | C | 0.283 | 0.012 | 0.001 | 2.90E-16 | 144 | 0.040 | 0.027 | 0.142 |
| 128041828 | 9 | rs13299559 | T | C | 0.450 | -0.010 | 0.001 | 7.10E-15 | 100 | -0.029 | 0.023 | 0.207 |
| 218146818 | 2 | rs13430869 | T | G | 0.742 | 0.013 | 0.001 | 8.00E-18 | 169 | -0.006 | 0.024 | 0.802 |
| 66351826 | 12 | rs1351394 | C | T | 0.512 | -0.025 | 0.001 | 9.30E-84 | 625 | -0.014 | 0.023 | 0.531 |
| 85818273 | 2 | rs1374370 | A | G | 0.305 | 0.010 | 0.001 | 4.00E-13 | 100 | -0.008 | 0.027 | 0.767 |
| 6542634 | 20 | rs1407031 | T | C | 0.397 | -0.008 | 0.001 | 1.90E-09 | 64 | 0.023 | 0.023 | 0.312 |
| 28410683 | 9 | rs1412234 | C | T | 0.327 | 0.012 | 0.001 | 4.80E-19 | 144 | -0.002 | 0.024 | 0.919 |
| 44752568 | 6 | rs1418433 | A | G | 0.588 | -0.008 | 0.001 | 7.00E-10 | 64 | 0.043 | 0.023 | 0.062 |
| 131136401 | 10 | rs1421035 | T | C | 0.348 | 0.008 | 0.001 | 2.60E-09 | 64 | -0.019 | 0.024 | 0.431 |
| 34025756 | 20 | rs143384 | G | A | 0.404 | 0.034 | 0.001 | 8.59E-146 | 1156 | 0.036 | 0.023 | 0.118 |
| 82174165 | 4 | rs1443536 | G | A | 0.305 | 0.012 | 0.001 | 2.70E-18 | 144 | 0.004 | 0.024 | 0.863 |
| 205388729 | 2 | rs1447498 | G | A | 0.770 | -0.009 | 0.002 | 3.90E-09 | 20 | 0.004 | 0.027 | 0.895 |
| 35659519 | 14 | rs145391487 | A | G | 0.108 | -0.013 | 0.002 | 2.60E-10 | 42 | 0.079 | 0.037 | 0.030 |
| 50377910 | 13 | rs146851424 | C | A | 0.022 | 0.059 | 0.005 | 5.40E-39 | 139 | 0.121 | 0.085 | 0.152 |
| 55993436 | 19 | rs147110934 | T | G | 0.024 | -0.030 | 0.004 | 1.20E-12 | 56 | -0.048 | 0.150 | 0.752 |
| 44028047 | 15 | rs147233090 | T | C | 0.025 | -0.028 | 0.004 | 5.10E-11 | 49 | -0.111 | 0.157 | 0.479 |
| 228721530 | 1 | rs148662000 | T | G | 0.064 | -0.015 | 0.003 | 1.90E-08 | 25 | 0.069 | 0.054 | 0.205 |
| 79412399 | 15 | rs1521624 | A | C | 0.471 | -0.010 | 0.001 | 7.90E-14 | 100 | -0.018 | 0.023 | 0.431 |
| 113035833 | 7 | rs1524445 | T | C | 0.421 | -0.011 | 0.001 | 1.50E-15 | 121 | 0.020 | 0.024 | 0.393 |
| 223963874 | 2 | rs1542224 | C | T | 0.719 | 0.013 | 0.001 | 3.00E-18 | 169 | 0.020 | 0.026 | 0.440 |
| 115036831 | 4 | rs1553065 | A | G | 0.430 | -0.008 | 0.001 | 1.30E-09 | 64 | -0.003 | 0.023 | 0.896 |
| 99186488 | 15 | rs1573891 | C | G | 0.158 | -0.021 | 0.002 | 1.70E-31 | 110 | 0.054 | 0.044 | 0.217 |
| 122657199 | 5 | rs1582931 | A | G | 0.473 | -0.019 | 0.001 | 6.90E-47 | 361 | 0.018 | 0.023 | 0.427 |
| 2270536 | 16 | rs1640779 | A | G | 0.453 | -0.013 | 0.001 | 1.20E-23 | 169 | -0.016 | 0.024 | 0.503 |
| 34837139 | 10 | rs1657222 | A | G | 0.396 | 0.009 | 0.001 | 1.90E-10 | 81 | -0.052 | 0.023 | 0.022 |
| 95566270 | 8 | rs16916881 | A | C | 0.235 | -0.011 | 0.002 | 1.30E-12 | 30 | 0.001 | 0.024 | 0.980 |
| 89383854 | 15 | rs16942324 | A | C | 0.027 | -0.037 | 0.004 | 9.30E-20 | 86 | -0.117 | 0.107 | 0.274 |
| 15816236 | 20 | rs16996657 | C | T | 0.128 | 0.012 | 0.002 | 4.90E-10 | 36 | 0.029 | 0.028 | 0.301 |
| 86723103 | 4 | rs17010961 | A | T | 0.139 | -0.012 | 0.002 | 7.50E-10 | 36 | -0.062 | 0.032 | 0.056 |
| 110154688 | 1 | rs17024393 | C | T | 0.026 | 0.033 | 0.004 | 2.30E-15 | 68 | 0.059 | 0.050 | 0.236 |
| 111604727 | 4 | rs17042011 | T | C | 0.207 | 0.009 | 0.002 | 2.50E-08 | 20 | -0.018 | 0.027 | 0.502 |
| 108917688 | 6 | rs17069647 | A | T | 0.094 | -0.013 | 0.002 | 1.60E-09 | 42 | -0.024 | 0.032 | 0.459 |
| 153358226 | 5 | rs17115481 | A | G | 0.270 | -0.010 | 0.001 | 9.10E-12 | 100 | 0.025 | 0.025 | 0.306 |
| 28779946 | 7 | rs17157112 | G | T | 0.471 | -0.008 | 0.001 | 9.00E-09 | 64 | -0.035 | 0.023 | 0.122 |
| 38104907 | 7 | rs1717726 | A | T | 0.651 | 0.008 | 0.001 | 4.30E-09 | 64 | -0.012 | 0.026 | 0.647 |
| 21894526 | 14 | rs17197114 | C | T | 0.177 | 0.011 | 0.002 | 4.60E-11 | 30 | 0.025 | 0.031 | 0.430 |
| 227259964 | 2 | rs17246129 | A | G | 0.305 | 0.011 | 0.001 | 2.20E-15 | 121 | 0.020 | 0.025 | 0.429 |
| 86823503 | 1 | rs17363646 | G | A | 0.136 | 0.013 | 0.002 | 6.00E-12 | 42 | 0.014 | 0.026 | 0.582 |
| 178565913 | 2 | rs17400325 | C | T | 0.042 | 0.021 | 0.003 | 4.80E-11 | 49 | -0.233 | 0.080 | 0.004 |
| 200402624 | 2 | rs17443541 | C | T | 0.189 | -0.011 | 0.002 | 1.40E-11 | 30 | 0.022 | 0.030 | 0.463 |
| 87631532 | 4 | rs17454077 | G | A | 0.036 | 0.025 | 0.004 | 1.60E-12 | 39 | 0.230 | 0.110 | 0.037 |
| 39672545 | 1 | rs17491275 | G | T | 0.160 | 0.017 | 0.002 | 7.40E-21 | 72 | 0.012 | 0.030 | 0.701 |
| 35758359 | 20 | rs1780673 | A | G | 0.811 | 0.013 | 0.002 | 7.70E-15 | 42 | -0.020 | 0.025 | 0.419 |
| 73454767 | 8 | rs17828687 | A | C | 0.573 | -0.011 | 0.001 | 1.00E-16 | 121 | -0.009 | 0.023 | 0.683 |
| 89301382 | 11 | rs1813212 | G | A | 0.446 | -0.009 | 0.001 | 1.50E-12 | 81 | -0.001 | 0.023 | 0.952 |
| 111874551 | 2 | rs1837367 | A | G | 0.483 | 0.009 | 0.001 | 3.50E-12 | 81 | 0.042 | 0.023 | 0.062 |
| 4667302 | 10 | rs1846221 | C | G | 0.175 | -0.010 | 0.002 | 1.00E-08 | 25 | -0.028 | 0.030 | 0.348 |
| 147086268 | 3 | rs1910466 | C | T | 0.498 | -0.008 | 0.001 | 3.30E-10 | 64 | -0.013 | 0.023 | 0.559 |
| 90063846 | 1 | rs1923766 | G | T | 0.244 | -0.008 | 0.002 | 3.00E-08 | 16 | -0.004 | 0.028 | 0.896 |
| 78443297 | 13 | rs1924936 | A | T | 0.774 | 0.015 | 0.002 | 1.10E-22 | 56 | 0.082 | 0.030 | 0.006 |
| 16461905 | 9 | rs1927635 | C | T | 0.354 | 0.010 | 0.001 | 3.10E-12 | 100 | -0.016 | 0.023 | 0.489 |
| 31251276 | 18 | rs1941697 | A | G | 0.449 | 0.009 | 0.001 | 7.00E-11 | 81 | 0.016 | 0.023 | 0.489 |
| 6279958 | 8 | rs1966818 | A | G | 0.172 | 0.010 | 0.002 | 1.60E-08 | 25 | -0.044 | 0.037 | 0.235 |
| 98368761 | 9 | rs1984119 | C | T | 0.256 | -0.013 | 0.002 | 1.60E-18 | 42 | 0.009 | 0.025 | 0.724 |
| 61996255 | 17 | rs2005172 | C | A | 0.640 | 0.021 | 0.001 | 9.50E-53 | 441 | 0.026 | 0.023 | 0.270 |
| 108023965 | 3 | rs2016469 | A | G | 0.372 | 0.009 | 0.001 | 3.30E-10 | 81 | -0.009 | 0.024 | 0.704 |
| 41883096 | 12 | rs2034923 | C | A | 0.489 | 0.010 | 0.001 | 3.00E-13 | 100 | 0.032 | 0.023 | 0.164 |
| 120435908 | 8 | rs2071519 | G | A | 0.250 | -0.012 | 0.002 | 1.60E-16 | 36 | -0.049 | 0.029 | 0.093 |
| 96071561 | 10 | rs2077218 | A | G | 0.762 | -0.013 | 0.002 | 4.30E-16 | 42 | -0.027 | 0.031 | 0.379 |
| 9033462 | 20 | rs2093147 | C | T | 0.643 | -0.008 | 0.001 | 2.20E-08 | 64 | 0.004 | 0.023 | 0.852 |
| 106216667 | 4 | rs2101975 | G | A | 0.432 | -0.016 | 0.001 | 5.20E-34 | 256 | 0.000 | 0.023 | 0.989 |
| 52818664 | 4 | rs2102278 | G | A | 0.323 | 0.011 | 0.001 | 1.40E-15 | 121 | 0.019 | 0.024 | 0.435 |
| 25939161 | 7 | rs2122823 | T | C | 0.211 | 0.010 | 0.002 | 1.10E-10 | 25 | -0.008 | 0.027 | 0.753 |
| 21584941 | 1 | rs212526 | C | T | 0.601 | 0.009 | 0.001 | 4.80E-12 | 81 | -0.023 | 0.024 | 0.328 |
| 169706079 | 2 | rs2140046 | C | T | 0.364 | -0.011 | 0.001 | 4.90E-17 | 121 | 0.000 | 0.023 | 0.996 |
| 233687080 | 2 | rs2197563 | A | G | 0.595 | 0.010 | 0.001 | 3.50E-14 | 100 | -0.014 | 0.026 | 0.572 |
| 51127270 | 13 | rs2225226 | T | C | 0.216 | -0.029 | 0.002 | 5.10E-72 | 210 | -0.002 | 0.026 | 0.930 |
| 124826462 | 12 | rs2229840 | T | C | 0.160 | 0.020 | 0.002 | 6.00E-30 | 100 | 0.029 | 0.032 | 0.372 |
| 12912629 | 4 | rs223942 | G | A | 0.615 | -0.008 | 0.001 | 2.00E-09 | 64 | -0.003 | 0.023 | 0.886 |
| 64491549 | 10 | rs224048 | A | G | 0.550 | -0.007 | 0.001 | 3.50E-08 | 49 | -0.016 | 0.023 | 0.492 |
| 8124315 | 1 | rs225111 | A | C | 0.432 | -0.008 | 0.001 | 1.80E-10 | 64 | -0.014 | 0.024 | 0.559 |
| 21223663 | 20 | rs2252720 | T | C | 0.674 | -0.013 | 0.001 | 2.70E-19 | 169 | 0.014 | 0.025 | 0.587 |
| 9975386 | 3 | rs2270894 | G | C | 0.203 | -0.017 | 0.002 | 2.90E-25 | 72 | 0.009 | 0.026 | 0.730 |
| 63430340 | 18 | rs2276190 | A | G | 0.277 | 0.009 | 0.001 | 1.00E-09 | 81 | 0.069 | 0.023 | 0.003 |
| 1665702 | 1 | rs2281175 | C | T | 0.405 | 0.011 | 0.001 | 2.00E-16 | 121 | -0.016 | 0.023 | 0.486 |
| 108090518 | 12 | rs2287214 | G | A | 0.398 | 0.007 | 0.001 | 2.40E-08 | 49 | -0.020 | 0.023 | 0.390 |
| 140244627 | 7 | rs2293176 | A | G | 0.334 | 0.009 | 0.001 | 3.10E-10 | 81 | 0.031 | 0.024 | 0.182 |
| 116522025 | 8 | rs2293888 | T | C | 0.218 | -0.017 | 0.002 | 8.10E-26 | 72 | 0.019 | 0.024 | 0.427 |
| 65520246 | 14 | rs2296316 | C | T | 0.465 | -0.010 | 0.001 | 4.20E-14 | 100 | -0.006 | 0.023 | 0.789 |
| 70531366 | 16 | rs2303792 | T | C | 0.359 | -0.008 | 0.001 | 2.90E-08 | 64 | -0.019 | 0.025 | 0.464 |
| 75003678 | 5 | rs2307111 | C | T | 0.395 | -0.019 | 0.001 | 1.10E-44 | 361 | -0.022 | 0.023 | 0.348 |
| 68203915 | 17 | rs236587 | C | T | 0.737 | -0.009 | 0.002 | 8.80E-09 | 20 | -0.062 | 0.025 | 0.013 |
| 99778226 | 10 | rs2439823 | G | A | 0.546 | 0.009 | 0.001 | 3.50E-11 | 81 | 0.031 | 0.023 | 0.169 |
| 176509193 | 5 | rs244711 | T | C | 0.686 | 0.017 | 0.002 | 5.80E-28 | 72 | 0.009 | 0.025 | 0.704 |
| 131447104 | 5 | rs247008 | G | A | 0.670 | 0.013 | 0.001 | 1.90E-20 | 169 | -0.018 | 0.023 | 0.437 |
| 67503501 | 17 | rs2521349 | A | G | 0.385 | 0.008 | 0.001 | 1.30E-08 | 64 | 0.015 | 0.023 | 0.527 |
| 5496480 | 5 | rs252938 | T | C | 0.649 | -0.009 | 0.001 | 4.00E-11 | 81 | -0.015 | 0.023 | 0.518 |
| 2859847 | 7 | rs2533879 | A | G | 0.300 | -0.021 | 0.001 | 1.30E-50 | 441 | -0.046 | 0.024 | 0.051 |
| 171224403 | 5 | rs254963 | G | A | 0.447 | -0.008 | 0.001 | 3.20E-10 | 64 | 0.003 | 0.023 | 0.893 |
| 72765116 | 1 | rs2568958 | A | G | 0.604 | 0.011 | 0.001 | 7.80E-17 | 121 | 0.001 | 0.024 | 0.965 |
| 12926096 | 3 | rs2569993 | C | T | 0.320 | 0.009 | 0.001 | 1.50E-10 | 81 | -0.006 | 0.024 | 0.802 |
| 90835589 | 9 | rs2578246 | A | G | 0.809 | -0.010 | 0.002 | 1.00E-09 | 25 | -0.034 | 0.031 | 0.275 |
| 95855576 | 5 | rs2611732 | G | A | 0.602 | 0.008 | 0.001 | 2.90E-09 | 64 | -0.050 | 0.025 | 0.042 |
| 225934295 | 1 | rs2615075 | G | A | 0.376 | 0.010 | 0.001 | 2.60E-14 | 100 | 0.031 | 0.024 | 0.184 |
| 88649369 | 4 | rs2627692 | T | C | 0.506 | -0.010 | 0.001 | 1.30E-13 | 100 | 0.004 | 0.023 | 0.848 |
| 6709838 | 20 | rs2650965 | G | A | 0.330 | -0.009 | 0.001 | 2.50E-10 | 81 | -0.002 | 0.024 | 0.930 |
| 201800511 | 1 | rs2678204 | G | T | 0.340 | 0.014 | 0.001 | 2.00E-23 | 196 | 0.016 | 0.025 | 0.519 |
| 3482554 | 4 | rs2699433 | T | C | 0.148 | -0.012 | 0.002 | 2.20E-11 | 36 | -0.020 | 0.036 | 0.593 |
| 34618937 | 6 | rs2744956 | C | T | 0.139 | 0.040 | 0.002 | 1.20E-102 | 400 | 0.064 | 0.029 | 0.026 |
| 35686564 | 9 | rs2756895 | T | C | 0.518 | 0.008 | 0.001 | 3.80E-10 | 64 | 0.030 | 0.023 | 0.185 |
| 28835463 | 10 | rs2772435 | A | G | 0.799 | -0.009 | 0.002 | 1.90E-08 | 20 | -0.120 | 0.029 | 0.000 |
| 235508270 | 1 | rs2789366 | A | G | 0.346 | -0.009 | 0.001 | 1.00E-11 | 81 | -0.025 | 0.024 | 0.283 |
| 49217261 | 19 | rs281385 | G | A | 0.879 | -0.012 | 0.002 | 7.30E-09 | 36 | 0.017 | 0.030 | 0.568 |
| 10734800 | 1 | rs284315 | G | A | 0.498 | -0.008 | 0.001 | 2.60E-09 | 64 | -0.022 | 0.023 | 0.346 |
| 41932275 | 19 | rs284662 | C | T | 0.620 | -0.010 | 0.001 | 1.50E-12 | 100 | 0.006 | 0.024 | 0.801 |
| 38399816 | 1 | rs28605759 | A | G | 0.454 | -0.009 | 0.001 | 2.90E-11 | 81 | -0.046 | 0.023 | 0.043 |
| 98216876 | 9 | rs28620532 | G | A | 0.349 | 0.017 | 0.001 | 7.70E-35 | 289 | 0.012 | 0.024 | 0.603 |
| 70106061 | 7 | rs2866719 | T | C | 0.369 | 0.009 | 0.001 | 9.50E-12 | 81 | 0.021 | 0.024 | 0.389 |
| 46763686 | 12 | rs2897968 | A | G | 0.607 | 0.015 | 0.001 | 6.40E-28 | 225 | 0.015 | 0.023 | 0.517 |
| 11878464 | 12 | rs2900208 | A | C | 0.354 | 0.013 | 0.001 | 8.50E-23 | 169 | 0.000 | 0.023 | 0.993 |
| 112138888 | 5 | rs2952615 | C | G | 0.618 | -0.013 | 0.001 | 1.20E-21 | 169 | 0.024 | 0.023 | 0.293 |
| 34299865 | 19 | rs29946 | C | T | 0.557 | 0.008 | 0.001 | 7.00E-09 | 64 | 0.049 | 0.023 | 0.033 |
| 77453226 | 12 | rs310796 | T | G | 0.681 | 0.011 | 0.001 | 6.20E-16 | 121 | -0.023 | 0.026 | 0.372 |
| 27917771 | 17 | rs3110496 | G | A | 0.686 | 0.009 | 0.001 | 9.00E-10 | 81 | -0.046 | 0.023 | 0.051 |
| 233074205 | 2 | rs3116201 | A | G | 0.098 | -0.018 | 0.002 | 1.40E-16 | 81 | 0.019 | 0.053 | 0.722 |
| 134363145 | 5 | rs31211 | A | G | 0.251 | -0.012 | 0.002 | 1.30E-16 | 36 | 0.022 | 0.026 | 0.389 |
| 24804088 | 14 | rs3212260 | T | A | 0.258 | 0.013 | 0.001 | 6.40E-19 | 169 | 0.029 | 0.027 | 0.289 |
| 67166731 | 16 | rs34017457 | A | G | 0.007 | 0.054 | 0.008 | 9.00E-12 | 46 | -0.001 | 0.149 | 0.994 |
| 64460104 | 17 | rs34055910 | G | A | 0.379 | -0.008 | 0.001 | 2.50E-08 | 64 | 0.013 | 0.023 | 0.580 |
| 68081246 | 16 | rs34147411 | T | C | 0.146 | 0.013 | 0.002 | 5.00E-12 | 42 | -0.040 | 0.033 | 0.220 |
| 159982551 | 4 | rs34227797 | C | G | 0.310 | 0.009 | 0.001 | 4.10E-10 | 81 | 0.022 | 0.025 | 0.378 |
| 22460204 | 8 | rs34268501 | G | A | 0.337 | -0.008 | 0.001 | 5.10E-09 | 64 | -0.006 | 0.025 | 0.794 |
| 20432033 | 3 | rs34373881 | A | G | 0.275 | -0.008 | 0.001 | 2.30E-08 | 64 | -0.013 | 0.026 | 0.614 |
| 78450517 | 1 | rs34517439 | A | C | 0.122 | 0.040 | 0.002 | 3.20E-86 | 400 | 0.074 | 0.034 | 0.030 |
| 98665549 | 3 | rs34693680 | T | C | 0.132 | 0.014 | 0.002 | 1.80E-13 | 49 | 0.009 | 0.035 | 0.788 |
| 23513093 | 7 | rs34776209 | T | C | 0.247 | -0.016 | 0.002 | 4.90E-27 | 64 | 0.001 | 0.028 | 0.979 |
| 103328352 | 12 | rs34825238 | T | G | 0.255 | 0.009 | 0.002 | 4.30E-09 | 20 | 0.028 | 0.029 | 0.338 |
| 123828042 | 4 | rs34848742 | G | T | 0.788 | -0.015 | 0.002 | 9.70E-20 | 56 | 0.000 | 0.030 | 0.992 |
| 32300634 | 20 | rs34879158 | C | A | 0.263 | -0.021 | 0.001 | 5.10E-47 | 441 | -0.021 | 0.027 | 0.441 |
| 46991243 | 19 | rs35050648 | T | G | 0.236 | 0.009 | 0.002 | 7.80E-09 | 20 | -0.015 | 0.026 | 0.561 |
| 43878459 | 11 | rs35251247 | A | G | 0.292 | 0.013 | 0.001 | 4.70E-18 | 169 | 0.038 | 0.025 | 0.131 |
| 30048553 | 16 | rs35467921 | T | C | 0.400 | 0.023 | 0.001 | 2.10E-66 | 529 | 0.025 | 0.024 | 0.290 |
| 2165576 | 11 | rs35506085 | A | G | 0.185 | -0.020 | 0.002 | 6.20E-31 | 100 | -0.013 | 0.030 | 0.660 |
| 17625915 | 22 | rs35665085 | A | G | 0.056 | -0.016 | 0.003 | 2.50E-08 | 28 | -0.053 | 0.050 | 0.287 |
| 43604167 | 6 | rs35679149 | G | A | 0.027 | -0.026 | 0.004 | 1.90E-10 | 42 | 0.105 | 0.103 | 0.311 |
| 153885503 | 3 | rs357486 | C | T | 0.543 | 0.011 | 0.001 | 4.30E-17 | 121 | -0.037 | 0.023 | 0.109 |
| 67457698 | 15 | rs35874463 | G | A | 0.058 | 0.019 | 0.003 | 7.70E-12 | 40 | -0.038 | 0.059 | 0.525 |
| 79093822 | 17 | rs36000545 | G | A | 0.396 | -0.017 | 0.001 | 2.40E-34 | 289 | 0.028 | 0.024 | 0.246 |
| 52814709 | 3 | rs36012032 | A | C | 0.091 | 0.018 | 0.002 | 1.40E-15 | 81 | 0.085 | 0.041 | 0.038 |
| 21578007 | 14 | rs36100359 | A | G | 0.139 | -0.011 | 0.002 | 7.10E-09 | 30 | 0.015 | 0.032 | 0.644 |
| 77401152 | 5 | rs365352 | A | G | 0.244 | -0.016 | 0.002 | 7.30E-25 | 64 | -0.012 | 0.029 | 0.676 |
| 127549187 | 5 | rs36695 | A | G | 0.504 | -0.008 | 0.001 | 2.80E-10 | 64 | 0.036 | 0.023 | 0.116 |
| 49168798 | 12 | rs3730071 | A | C | 0.030 | -0.025 | 0.004 | 2.70E-11 | 39 | -0.131 | 0.113 | 0.246 |
| 70287303 | 10 | rs3740591 | T | C | 0.445 | 0.011 | 0.001 | 2.70E-16 | 121 | 0.010 | 0.024 | 0.666 |
| 38351626 | 17 | rs3744806 | G | C | 0.300 | 0.009 | 0.001 | 2.10E-10 | 81 | -0.035 | 0.024 | 0.139 |
| 56497903 | 12 | rs3759094 | T | C | 0.337 | -0.012 | 0.001 | 1.70E-17 | 144 | 0.031 | 0.024 | 0.198 |
| 22445991 | 1 | rs3765351 | T | C | 0.541 | -0.010 | 0.001 | 3.20E-13 | 100 | -0.016 | 0.023 | 0.472 |
| 39445385 | 7 | rs3778934 | C | A | 0.341 | -0.008 | 0.001 | 9.00E-10 | 64 | 0.001 | 0.024 | 0.983 |
| 17979793 | 7 | rs3800963 | A | G | 0.428 | -0.007 | 0.001 | 2.80E-08 | 49 | 0.012 | 0.023 | 0.591 |
| 47569003 | 19 | rs3810291 | A | G | 0.675 | 0.018 | 0.001 | 1.70E-38 | 324 | 0.001 | 0.024 | 0.958 |
| 139252879 | 9 | rs3812550 | G | A | 0.476 | 0.007 | 0.001 | 4.60E-08 | 49 | -0.013 | 0.023 | 0.554 |
| 139059017 | 5 | rs3822742 | A | C | 0.371 | 0.014 | 0.001 | 1.10E-26 | 196 | -0.011 | 0.024 | 0.644 |
| 75001480 | 1 | rs3845344 | T | C | 0.391 | 0.009 | 0.001 | 3.70E-12 | 81 | 0.005 | 0.023 | 0.823 |
| 152170247 | 6 | rs3853252 | A | G | 0.455 | 0.016 | 0.001 | 2.80E-33 | 256 | -0.006 | 0.023 | 0.780 |
| 141816555 | 5 | rs3853474 | T | C | 0.377 | -0.008 | 0.001 | 1.10E-09 | 64 | -0.004 | 0.023 | 0.880 |
| 38281658 | 8 | rs3925 | A | G | 0.245 | -0.010 | 0.002 | 4.80E-11 | 25 | 0.002 | 0.030 | 0.942 |
| 19839415 | 6 | rs41271299 | T | C | 0.051 | 0.043 | 0.003 | 7.10E-48 | 205 | -0.079 | 0.090 | 0.381 |
| 42070374 | 22 | rs41311445 | C | A | 0.096 | -0.025 | 0.002 | 3.20E-28 | 156 | 0.006 | 0.033 | 0.862 |
| 31001301 | 4 | rs4132132 | C | T | 0.444 | 0.009 | 0.001 | 4.50E-12 | 81 | -0.030 | 0.023 | 0.204 |
| 46929951 | 2 | rs41458449 | C | G | 0.144 | -0.011 | 0.002 | 1.40E-08 | 30 | 0.051 | 0.029 | 0.080 |
| 145839264 | 4 | rs4240326 | G | A | 0.550 | -0.022 | 0.001 | 5.00E-62 | 484 | -0.016 | 0.023 | 0.495 |
| 46615376 | 22 | rs4253755 | A | G | 0.128 | 0.011 | 0.002 | 7.80E-09 | 30 | 0.003 | 0.042 | 0.935 |
| 28645341 | 11 | rs4268495 | C | G | 0.374 | -0.009 | 0.001 | 1.90E-10 | 81 | -0.031 | 0.024 | 0.184 |
| 168256240 | 5 | rs4282339 | A | G | 0.208 | -0.018 | 0.002 | 2.00E-30 | 81 | -0.038 | 0.029 | 0.192 |
| 23166656 | 8 | rs4311660 | A | C | 0.725 | -0.010 | 0.001 | 1.30E-11 | 100 | -0.018 | 0.025 | 0.470 |
| 20735408 | 18 | rs4369779 | C | T | 0.789 | 0.026 | 0.002 | 2.50E-59 | 169 | 0.029 | 0.027 | 0.291 |
| 1866892 | 17 | rs4525525 | T | G | 0.264 | -0.011 | 0.002 | 1.20E-12 | 30 | -0.010 | 0.026 | 0.719 |
| 100814858 | 2 | rs4556997 | A | C | 0.136 | 0.014 | 0.002 | 1.50E-13 | 49 | -0.066 | 0.033 | 0.047 |
| 59204270 | 13 | rs4567604 | T | G | 0.193 | -0.010 | 0.002 | 7.10E-10 | 25 | 0.012 | 0.030 | 0.680 |
| 4879766 | 16 | rs4619406 | C | T | 0.498 | 0.011 | 0.001 | 7.90E-16 | 121 | 0.032 | 0.023 | 0.159 |
| 152310614 | 3 | rs4635681 | G | A | 0.156 | 0.011 | 0.002 | 1.90E-10 | 30 | -0.055 | 0.029 | 0.055 |
| 2200390 | 1 | rs4648626 | A | C | 0.457 | 0.008 | 0.001 | 1.10E-09 | 64 | -0.011 | 0.023 | 0.644 |
| 33776441 | 1 | rs4653016 | A | C | 0.683 | 0.015 | 0.001 | 4.30E-27 | 225 | -0.011 | 0.024 | 0.638 |
| 134468311 | 9 | rs4740292 | G | A | 0.834 | 0.011 | 0.002 | 2.20E-09 | 30 | 0.035 | 0.036 | 0.326 |
| 41570459 | 1 | rs475390 | A | G | 0.776 | -0.021 | 0.002 | 2.10E-40 | 110 | -0.028 | 0.027 | 0.304 |
| 68080886 | 15 | rs4776970 | T | A | 0.358 | -0.013 | 0.001 | 4.50E-20 | 169 | -0.064 | 0.024 | 0.007 |
| 19834422 | 16 | rs4782286 | A | G | 0.211 | -0.012 | 0.002 | 7.40E-15 | 36 | 0.048 | 0.030 | 0.113 |
| 46466927 | 21 | rs4819021 | C | T | 0.477 | -0.009 | 0.001 | 5.10E-11 | 81 | -0.033 | 0.023 | 0.148 |
| 172137343 | 3 | rs485554 | C | G | 0.315 | 0.016 | 0.001 | 1.20E-28 | 256 | 0.028 | 0.024 | 0.251 |
| 88254820 | 3 | rs4858940 | C | T | 0.886 | 0.018 | 0.002 | 3.10E-18 | 81 | -0.019 | 0.038 | 0.623 |
| 1745486 | 4 | rs4865465 | A | G | 0.535 | 0.010 | 0.001 | 2.10E-13 | 100 | -0.011 | 0.024 | 0.646 |
| 54882505 | 5 | rs4865956 | A | T | 0.697 | -0.011 | 0.001 | 9.70E-15 | 121 | 0.017 | 0.025 | 0.503 |
| 54084032 | 13 | rs4883723 | A | G | 0.140 | 0.017 | 0.002 | 1.70E-18 | 72 | 0.080 | 0.033 | 0.014 |
| 61003889 | 14 | rs4899012 | C | G | 0.607 | -0.021 | 0.001 | 6.60E-57 | 441 | 0.000 | 0.025 | 0.988 |
| 142730376 | 5 | rs4912905 | C | G | 0.249 | -0.010 | 0.002 | 2.30E-11 | 25 | 0.015 | 0.026 | 0.575 |
| 109003321 | 6 | rs4946936 | C | T | 0.713 | 0.022 | 0.001 | 1.60E-51 | 484 | -0.015 | 0.024 | 0.521 |
| 68158831 | 17 | rs4968799 | T | A | 0.112 | -0.018 | 0.002 | 1.90E-18 | 81 | 0.007 | 0.038 | 0.852 |
| 41222586 | 3 | rs4974072 | A | G | 0.490 | 0.011 | 0.001 | 1.70E-17 | 121 | -0.043 | 0.023 | 0.058 |
| 578349 | 12 | rs4980826 | A | C | 0.396 | 0.008 | 0.001 | 4.10E-09 | 64 | 0.019 | 0.024 | 0.420 |
| 217893504 | 1 | rs5017213 | C | T | 0.355 | 0.008 | 0.001 | 4.30E-09 | 64 | 0.013 | 0.024 | 0.572 |
| 51738352 | 6 | rs5019542 | T | C | 0.636 | -0.010 | 0.001 | 1.70E-12 | 100 | 0.008 | 0.023 | 0.729 |
| 111256812 | 5 | rs505575 | C | T | 0.673 | -0.010 | 0.001 | 5.30E-12 | 100 | -0.001 | 0.023 | 0.963 |
| 28212824 | 7 | rs508347 | C | T | 0.703 | -0.012 | 0.001 | 1.80E-16 | 144 | -0.053 | 0.024 | 0.027 |
| 36653677 | 1 | rs522468 | G | T | 0.715 | 0.008 | 0.001 | 2.30E-08 | 64 | 0.050 | 0.026 | 0.059 |
| 30165465 | 13 | rs532499 | C | T | 0.741 | -0.009 | 0.001 | 3.20E-10 | 81 | -0.002 | 0.024 | 0.934 |
| 177889480 | 1 | rs543874 | G | A | 0.205 | 0.029 | 0.002 | 1.40E-72 | 210 | -0.011 | 0.030 | 0.724 |
| 89435868 | 8 | rs55674305 | A | G | 0.306 | -0.011 | 0.001 | 9.40E-14 | 121 | -0.021 | 0.025 | 0.386 |
| 991306 | 12 | rs55726687 | A | G | 0.210 | 0.017 | 0.002 | 3.60E-26 | 72 | -0.006 | 0.028 | 0.828 |
| 171317318 | 5 | rs55758152 | A | G | 0.326 | 0.010 | 0.001 | 3.30E-12 | 100 | -0.017 | 0.024 | 0.493 |
| 7559037 | 17 | rs55831773 | T | C | 0.199 | -0.015 | 0.002 | 2.30E-20 | 56 | -0.034 | 0.033 | 0.299 |
| 45928049 | 18 | rs55854145 | C | A | 0.055 | -0.016 | 0.003 | 1.50E-08 | 28 | -0.003 | 0.058 | 0.966 |
| 53806453 | 16 | rs56094641 | G | A | 0.405 | 0.041 | 0.001 | 1.00E-200 | 1681 | 0.050 | 0.023 | 0.028 |
| 105906522 | 14 | rs56130943 | C | A | 0.233 | 0.009 | 0.002 | 1.50E-08 | 20 | -0.029 | 0.029 | 0.306 |
| 74097622 | 7 | rs56383938 | G | A | 0.087 | -0.015 | 0.002 | 3.50E-11 | 56 | 0.030 | 0.038 | 0.433 |
| 2656989 | 18 | rs57126421 | G | A | 0.238 | -0.011 | 0.002 | 1.50E-13 | 30 | 0.006 | 0.027 | 0.833 |
| 117267884 | 11 | rs573455 | G | A | 0.534 | 0.007 | 0.001 | 2.50E-08 | 49 | -0.006 | 0.023 | 0.786 |
| 30365780 | 22 | rs5752989 | A | G | 0.571 | -0.010 | 0.001 | 1.10E-14 | 100 | 0.015 | 0.023 | 0.506 |
| 58048295 | 18 | rs57636386 | C | T | 0.084 | -0.024 | 0.002 | 3.00E-24 | 144 | -0.065 | 0.052 | 0.215 |
| 50714289 | 22 | rs5771118 | C | T | 0.742 | 0.009 | 0.002 | 6.60E-10 | 20 | 0.000 | 0.028 | 0.997 |
| 81591034 | 6 | rs578366 | G | A | 0.420 | -0.010 | 0.001 | 2.10E-14 | 100 | 0.010 | 0.023 | 0.650 |
| 112249583 | 2 | rs58584712 | A | G | 0.211 | 0.009 | 0.002 | 9.30E-09 | 20 | 0.031 | 0.030 | 0.307 |
| 140439740 | 6 | rs599004 | T | C | 0.281 | -0.011 | 0.001 | 7.70E-14 | 121 | -0.018 | 0.028 | 0.527 |
| 56106928 | 2 | rs59985551 | T | C | 0.226 | -0.019 | 0.002 | 2.80E-35 | 90 | -0.056 | 0.027 | 0.040 |
| 172098794 | 1 | rs60077625 | A | G | 0.314 | 0.014 | 0.001 | 3.80E-24 | 196 | 0.057 | 0.026 | 0.027 |
| 57463472 | 20 | rs6026578 | G | C | 0.625 | -0.010 | 0.001 | 4.10E-14 | 100 | 0.022 | 0.024 | 0.356 |
| 44495988 | 20 | rs6130953 | G | A | 0.624 | 0.008 | 0.001 | 1.10E-08 | 64 | 0.004 | 0.024 | 0.865 |
| 20058992 | 20 | rs6136938 | A | G | 0.436 | -0.009 | 0.001 | 8.00E-13 | 81 | 0.029 | 0.023 | 0.202 |
| 32544327 | 20 | rs6142059 | C | T | 0.492 | 0.010 | 0.001 | 1.80E-15 | 100 | -0.013 | 0.023 | 0.576 |
| 95515900 | 9 | rs61628776 | G | A | 0.141 | -0.015 | 0.002 | 1.70E-15 | 56 | -0.017 | 0.037 | 0.639 |
| 77761919 | 8 | rs61729527 | T | C | 0.052 | -0.024 | 0.003 | 1.30E-15 | 64 | -0.019 | 0.046 | 0.687 |
| 173580303 | 1 | rs61828917 | T | C | 0.125 | 0.011 | 0.002 | 2.70E-08 | 30 | -0.051 | 0.037 | 0.165 |
| 2813345 | 11 | rs61869763 | T | C | 0.105 | 0.018 | 0.002 | 3.40E-16 | 81 | 0.092 | 0.040 | 0.021 |
| 101531854 | 14 | rs61992671 | G | A | 0.492 | -0.011 | 0.001 | 1.50E-16 | 121 | 0.033 | 0.023 | 0.153 |
| 9498143 | 3 | rs62246311 | A | G | 0.102 | 0.015 | 0.002 | 6.90E-12 | 56 | 0.024 | 0.046 | 0.612 |
| 95728898 | 5 | rs6235 | G | C | 0.268 | 0.017 | 0.001 | 4.90E-30 | 289 | 0.024 | 0.025 | 0.337 |
| 42724294 | 5 | rs62372052 | G | A | 0.110 | 0.026 | 0.002 | 1.40E-35 | 169 | -0.002 | 0.035 | 0.953 |
| 26180634 | 6 | rs62396185 | C | G | 0.260 | -0.023 | 0.001 | 2.90E-55 | 529 | -0.018 | 0.023 | 0.446 |
| 166592164 | 6 | rs62439025 | C | G | 0.152 | 0.012 | 0.002 | 2.20E-10 | 36 | -0.003 | 0.037 | 0.936 |
| 44892742 | 7 | rs62460522 | T | C | 0.070 | 0.015 | 0.003 | 1.20E-08 | 25 | 0.038 | 0.066 | 0.565 |
| 92716556 | 7 | rs62466118 | A | G | 0.028 | -0.025 | 0.004 | 1.50E-09 | 39 | 0.008 | 0.125 | 0.951 |
| 57160328 | 8 | rs62515437 | T | G | 0.225 | 0.018 | 0.002 | 3.00E-32 | 81 | 0.019 | 0.025 | 0.455 |
| 8670147 | 19 | rs62621197 | T | C | 0.037 | -0.036 | 0.004 | 1.20E-23 | 81 | -0.150 | 0.081 | 0.063 |
| 101718239 | 15 | rs62621400 | G | C | 0.058 | -0.021 | 0.003 | 4.60E-14 | 49 | -0.064 | 0.048 | 0.183 |
| 127015083 | 7 | rs62621812 | A | G | 0.020 | 0.040 | 0.005 | 2.10E-17 | 64 | -0.059 | 0.057 | 0.304 |
| 242608984 | 2 | rs6437277 | G | A | 0.769 | -0.012 | 0.002 | 1.00E-14 | 36 | -0.038 | 0.027 | 0.166 |
| 55656840 | 15 | rs6493780 | G | A | 0.093 | -0.015 | 0.002 | 1.70E-11 | 56 | -0.003 | 0.041 | 0.936 |
| 49778147 | 16 | rs6500249 | G | A | 0.735 | -0.009 | 0.001 | 5.10E-09 | 81 | 0.008 | 0.029 | 0.773 |
| 190287713 | 1 | rs655598 | A | G | 0.563 | -0.010 | 0.001 | 1.80E-14 | 100 | -0.008 | 0.023 | 0.743 |
| 40766662 | 13 | rs6563808 | C | T | 0.734 | -0.009 | 0.001 | 1.90E-10 | 81 | -0.019 | 0.024 | 0.440 |
| 57829135 | 18 | rs6567160 | C | T | 0.233 | 0.046 | 0.002 | 2.80E-193 | 529 | 0.027 | 0.029 | 0.364 |
| 142716286 | 6 | rs6570509 | T | G | 0.287 | -0.014 | 0.001 | 1.60E-23 | 196 | 0.004 | 0.026 | 0.866 |
| 94023972 | 14 | rs6575340 | A | G | 0.636 | 0.010 | 0.001 | 2.10E-12 | 100 | -0.003 | 0.024 | 0.893 |
| 124165615 | 10 | rs6585827 | A | G | 0.471 | 0.012 | 0.001 | 4.20E-20 | 144 | 0.003 | 0.024 | 0.886 |
| 69449076 | 11 | rs667515 | C | G | 0.386 | -0.011 | 0.001 | 1.30E-15 | 121 | -0.017 | 0.024 | 0.485 |
| 214659762 | 1 | rs6675441 | A | G | 0.234 | -0.012 | 0.002 | 1.70E-15 | 36 | -0.047 | 0.030 | 0.125 |
| 56583274 | 1 | rs6681795 | G | A | 0.228 | 0.015 | 0.002 | 1.50E-21 | 56 | 0.080 | 0.027 | 0.003 |
| 42805360 | 13 | rs67141907 | T | C | 0.149 | 0.012 | 0.002 | 3.80E-10 | 36 | 0.048 | 0.037 | 0.187 |
| 629510 | 2 | rs6743060 | A | C | 0.828 | 0.037 | 0.002 | 1.20E-101 | 342 | 0.028 | 0.031 | 0.356 |
| 3393100 | 12 | rs67551338 | T | C | 0.061 | 0.023 | 0.003 | 1.30E-17 | 59 | -0.068 | 0.058 | 0.246 |
| 128992047 | 3 | rs6762578 | A | G | 0.778 | 0.016 | 0.002 | 3.60E-23 | 64 | 0.038 | 0.030 | 0.218 |
| 56686329 | 3 | rs6762851 | C | T | 0.357 | -0.012 | 0.001 | 3.30E-18 | 144 | -0.017 | 0.023 | 0.469 |
| 62376645 | 3 | rs6777784 | T | G | 0.617 | 0.008 | 0.001 | 6.80E-09 | 64 | 0.003 | 0.024 | 0.917 |
| 85663849 | 3 | rs6779752 | A | G | 0.638 | -0.013 | 0.001 | 5.50E-22 | 169 | -0.019 | 0.025 | 0.450 |
| 49995518 | 3 | rs6792892 | C | T | 0.531 | 0.015 | 0.001 | 8.80E-31 | 225 | -0.006 | 0.023 | 0.785 |
| 47065115 | 17 | rs68106312 | A | G | 0.096 | 0.019 | 0.002 | 4.60E-18 | 90 | -0.014 | 0.037 | 0.702 |
| 172753555 | 5 | rs6874142 | G | T | 0.114 | 0.018 | 0.002 | 1.90E-17 | 81 | -0.003 | 0.040 | 0.934 |
| 76639839 | 7 | rs6951489 | G | A | 0.825 | -0.017 | 0.002 | 1.40E-21 | 72 | -0.016 | 0.028 | 0.567 |
| 53385529 | 5 | rs695922 | G | A | 0.837 | -0.010 | 0.002 | 4.50E-08 | 25 | -0.090 | 0.031 | 0.003 |
| 144993324 | 8 | rs6984820 | T | C | 0.422 | -0.010 | 0.001 | 1.60E-13 | 100 | 0.004 | 0.023 | 0.873 |
| 88937617 | 9 | rs700768 | A | T | 0.335 | 0.009 | 0.001 | 2.30E-10 | 81 | 0.045 | 0.023 | 0.052 |
| 119129257 | 9 | rs7033487 | C | T | 0.198 | -0.022 | 0.002 | 4.60E-42 | 121 | 0.001 | 0.027 | 0.971 |
| 103064247 | 12 | rs703593 | G | A | 0.482 | -0.012 | 0.001 | 1.00E-18 | 144 | 0.001 | 0.023 | 0.962 |
| 93180531 | 11 | rs7109581 | G | T | 0.420 | 0.007 | 0.001 | 4.30E-08 | 49 | 0.020 | 0.024 | 0.406 |
| 133658661 | 11 | rs7111235 | C | T | 0.494 | 0.008 | 0.001 | 7.30E-10 | 64 | 0.008 | 0.024 | 0.729 |
| 68388220 | 11 | rs7129320 | A | G | 0.166 | -0.023 | 0.002 | 3.30E-38 | 132 | 0.045 | 0.040 | 0.261 |
| 50263148 | 12 | rs7132908 | A | G | 0.384 | 0.018 | 0.001 | 2.20E-42 | 324 | 0.036 | 0.023 | 0.121 |
| 24071748 | 12 | rs7134283 | A | G | 0.283 | -0.012 | 0.001 | 4.40E-17 | 144 | -0.015 | 0.025 | 0.547 |
| 2160503 | 16 | rs71385734 | G | T | 0.170 | -0.024 | 0.002 | 2.70E-42 | 144 | 0.031 | 0.030 | 0.297 |
| 79899454 | 14 | rs7141420 | T | C | 0.516 | 0.015 | 0.001 | 9.60E-29 | 225 | 0.042 | 0.023 | 0.071 |
| 144024781 | 2 | rs71423263 | G | T | 0.141 | 0.013 | 0.002 | 8.20E-13 | 42 | -0.031 | 0.037 | 0.404 |
| 81660642 | 16 | rs7188009 | A | G | 0.404 | 0.009 | 0.001 | 1.10E-10 | 81 | -0.041 | 0.026 | 0.115 |
| 29211667 | 17 | rs7223535 | A | G | 0.270 | -0.025 | 0.001 | 1.90E-63 | 625 | -0.041 | 0.025 | 0.102 |
| 46516468 | 18 | rs7229520 | A | G | 0.662 | -0.010 | 0.001 | 3.70E-13 | 100 | -0.016 | 0.024 | 0.505 |
| 46577056 | 7 | rs723149 | G | A | 0.563 | -0.011 | 0.001 | 3.30E-17 | 121 | 0.035 | 0.023 | 0.123 |
| 141105570 | 3 | rs724016 | G | A | 0.444 | 0.031 | 0.001 | 6.70E-120 | 961 | 0.004 | 0.023 | 0.869 |
| 30710410 | 19 | rs7245985 | G | T | 0.208 | -0.011 | 0.002 | 4.00E-12 | 30 | -0.028 | 0.029 | 0.342 |
| 51530167 | 15 | rs726547 | A | G | 0.046 | -0.021 | 0.003 | 1.60E-11 | 49 | -0.005 | 0.044 | 0.916 |
| 57122215 | 8 | rs72656010 | C | T | 0.132 | -0.036 | 0.002 | 1.10E-75 | 324 | -0.041 | 0.034 | 0.236 |
| 184225295 | 4 | rs72703409 | C | G | 0.066 | -0.015 | 0.003 | 1.50E-08 | 25 | -0.057 | 0.059 | 0.337 |
| 129186110 | 8 | rs72722756 | C | T | 0.180 | 0.010 | 0.002 | 1.50E-08 | 25 | 0.057 | 0.035 | 0.104 |
| 53508802 | 16 | rs72801843 | A | T | 0.301 | 0.016 | 0.001 | 7.40E-30 | 256 | -0.005 | 0.026 | 0.843 |
| 172416376 | 2 | rs72885917 | C | A | 0.247 | -0.020 | 0.002 | 4.10E-41 | 100 | -0.025 | 0.029 | 0.384 |
| 50816887 | 6 | rs72892910 | T | G | 0.172 | 0.023 | 0.002 | 5.40E-41 | 132 | 0.007 | 0.028 | 0.809 |
| 19717056 | 19 | rs73004967 | G | A | 0.069 | -0.017 | 0.003 | 1.70E-11 | 32 | -0.001 | 0.047 | 0.990 |
| 94083105 | 12 | rs7301341 | C | T | 0.327 | -0.010 | 0.001 | 8.00E-12 | 100 | 0.013 | 0.024 | 0.593 |
| 164126233 | 6 | rs73013411 | A | C | 0.131 | 0.014 | 0.002 | 2.70E-13 | 49 | 0.021 | 0.042 | 0.611 |
| 185828465 | 3 | rs73052033 | C | T | 0.185 | -0.016 | 0.002 | 1.10E-22 | 64 | 0.015 | 0.031 | 0.620 |
| 50658010 | 5 | rs73093103 | T | A | 0.034 | 0.022 | 0.004 | 6.20E-10 | 30 | 0.049 | 0.065 | 0.455 |
| 102388920 | 12 | rs7312646 | C | A | 0.521 | -0.010 | 0.001 | 6.80E-15 | 100 | 0.021 | 0.023 | 0.352 |
| 185490184 | 3 | rs73175572 | G | A | 0.112 | 0.028 | 0.002 | 1.00E-41 | 196 | -0.012 | 0.035 | 0.742 |
| 17383170 | 21 | rs73189390 | A | G | 0.184 | -0.010 | 0.002 | 1.50E-08 | 25 | 0.011 | 0.029 | 0.704 |
| 97034410 | 13 | rs7321045 | A | G | 0.449 | 0.009 | 0.001 | 3.30E-12 | 81 | 0.013 | 0.023 | 0.576 |
| 28489339 | 4 | rs73213484 | T | A | 0.141 | -0.014 | 0.002 | 5.10E-14 | 49 | -0.058 | 0.032 | 0.070 |
| 18549889 | 10 | rs73601548 | T | C | 0.115 | 0.015 | 0.002 | 4.40E-13 | 56 | 0.054 | 0.046 | 0.246 |
| 61564901 | 20 | rs73619441 | G | T | 0.144 | -0.013 | 0.002 | 8.40E-12 | 42 | -0.061 | 0.036 | 0.091 |
| 99495351 | 15 | rs74032128 | G | A | 0.033 | 0.024 | 0.004 | 7.30E-11 | 36 | 0.114 | 0.083 | 0.167 |
| 2093603 | 11 | rs74048171 | A | C | 0.258 | -0.011 | 0.001 | 1.60E-12 | 121 | -0.007 | 0.027 | 0.791 |
| 69929677 | 11 | rs744205 | A | G | 0.544 | 0.011 | 0.001 | 3.00E-16 | 121 | -0.005 | 0.023 | 0.819 |
| 74972138 | 18 | rs74494415 | T | C | 0.040 | -0.025 | 0.003 | 7.70E-14 | 69 | -0.047 | 0.043 | 0.279 |
| 159895536 | 1 | rs7513326 | A | G | 0.508 | -0.007 | 0.001 | 2.10E-08 | 49 | -0.014 | 0.023 | 0.535 |
| 66434743 | 1 | rs7519259 | A | G | 0.528 | 0.008 | 0.001 | 2.10E-09 | 64 | 0.049 | 0.023 | 0.033 |
| 74824970 | 2 | rs752070 | G | A | 0.126 | 0.012 | 0.002 | 2.90E-09 | 36 | 0.001 | 0.033 | 0.972 |
| 42305131 | 3 | rs754635 | G | C | 0.887 | 0.014 | 0.002 | 2.80E-11 | 49 | -0.043 | 0.038 | 0.251 |
| 50604019 | 7 | rs75581912 | A | G | 0.117 | 0.017 | 0.002 | 8.50E-18 | 72 | -0.017 | 0.039 | 0.659 |
| 59497277 | 17 | rs757608 | G | A | 0.670 | -0.015 | 0.001 | 4.50E-27 | 225 | -0.013 | 0.026 | 0.619 |
| 139734697 | 7 | rs757833 | A | C | 0.328 | 0.008 | 0.001 | 1.80E-08 | 64 | -0.042 | 0.025 | 0.093 |
| 99500978 | 9 | rs76067562 | T | G | 0.095 | 0.015 | 0.002 | 1.10E-11 | 56 | -0.104 | 0.044 | 0.018 |
| 31834769 | 6 | rs76116290 | G | C | 0.046 | -0.018 | 0.003 | 4.00E-09 | 36 | 0.035 | 0.054 | 0.516 |
| 30078634 | 3 | rs7612882 | A | G | 0.546 | 0.008 | 0.001 | 3.00E-09 | 64 | -0.018 | 0.023 | 0.434 |
| 61267398 | 3 | rs7613368 | C | T | 0.260 | -0.010 | 0.001 | 1.80E-10 | 100 | 0.030 | 0.025 | 0.219 |
| 25110415 | 3 | rs7619139 | A | T | 0.589 | 0.013 | 0.001 | 3.40E-21 | 169 | -0.002 | 0.023 | 0.922 |
| 169000888 | 6 | rs76307059 | G | C | 0.046 | -0.019 | 0.003 | 1.00E-09 | 40 | -0.050 | 0.049 | 0.306 |
| 72505534 | 16 | rs76513770 | C | T | 0.128 | -0.017 | 0.002 | 9.80E-18 | 72 | 0.010 | 0.031 | 0.751 |
| 17874089 | 4 | rs7671110 | T | C | 0.158 | -0.030 | 0.002 | 3.40E-63 | 225 | 0.008 | 0.039 | 0.831 |
| 154994978 | 1 | rs76798800 | T | G | 0.266 | 0.023 | 0.001 | 9.60E-53 | 529 | 0.082 | 0.027 | 0.002 |
| 180167906 | 4 | rs7683836 | A | G | 0.557 | -0.008 | 0.001 | 3.10E-09 | 64 | 0.001 | 0.023 | 0.976 |
| 4384844 | 12 | rs76895963 | G | T | 0.021 | 0.096 | 0.005 | 6.20E-80 | 369 | 0.098 | 0.068 | 0.148 |
| 79429575 | 17 | rs77093479 | G | C | 0.165 | -0.010 | 0.002 | 1.50E-08 | 25 | 0.086 | 0.030 | 0.005 |
| 430975 | 2 | rs77165542 | T | C | 0.035 | -0.064 | 0.004 | 3.10E-71 | 256 | 0.064 | 0.087 | 0.463 |
| 88411214 | 5 | rs7728690 | T | C | 0.386 | -0.010 | 0.001 | 4.70E-15 | 100 | -0.020 | 0.024 | 0.405 |
| 36181627 | 5 | rs7731023 | G | A | 0.574 | 0.008 | 0.001 | 7.70E-10 | 64 | 0.042 | 0.024 | 0.079 |
| 130374461 | 6 | rs7740107 | A | T | 0.736 | -0.029 | 0.001 | 1.00E-86 | 841 | 0.014 | 0.026 | 0.585 |
| 152339615 | 6 | rs7755185 | G | A | 0.311 | 0.009 | 0.001 | 6.90E-10 | 81 | -0.013 | 0.024 | 0.579 |
| 93241640 | 7 | rs7780752 | C | T | 0.360 | 0.013 | 0.001 | 2.40E-21 | 169 | -0.009 | 0.026 | 0.713 |
| 107971673 | 1 | rs77848106 | A | C | 0.296 | -0.010 | 0.001 | 6.20E-12 | 100 | -0.012 | 0.023 | 0.610 |
| 33260875 | 3 | rs78149371 | G | A | 0.061 | -0.015 | 0.003 | 2.20E-08 | 25 | -0.070 | 0.062 | 0.254 |
| 130719567 | 8 | rs7815955 | T | A | 0.203 | -0.015 | 0.002 | 8.20E-20 | 56 | -0.001 | 0.030 | 0.963 |
| 64773101 | 11 | rs78287937 | G | T | 0.090 | 0.013 | 0.002 | 8.40E-09 | 42 | -0.040 | 0.060 | 0.512 |
| 7571752 | 17 | rs78378222 | G | T | 0.012 | 0.081 | 0.006 | 2.00E-40 | 182 | 0.000 | 0.088 | 0.996 |
| 78107140 | 8 | rs7842996 | A | T | 0.284 | 0.019 | 0.001 | 6.80E-38 | 361 | -0.012 | 0.027 | 0.650 |
| 114693230 | 10 | rs7912286 | G | A | 0.602 | -0.009 | 0.001 | 7.50E-12 | 81 | 0.031 | 0.023 | 0.187 |
| 9476419 | 11 | rs7927350 | A | C | 0.494 | -0.010 | 0.001 | 1.10E-14 | 100 | -0.009 | 0.023 | 0.693 |
| 130796248 | 11 | rs7933085 | G | A | 0.508 | 0.008 | 0.001 | 1.30E-09 | 64 | -0.004 | 0.023 | 0.850 |
| 67024534 | 11 | rs7952436 | T | C | 0.082 | -0.035 | 0.002 | 8.10E-48 | 306 | -0.078 | 0.052 | 0.132 |
| 12827900 | 12 | rs7969505 | C | G | 0.114 | -0.013 | 0.002 | 1.40E-10 | 42 | -0.021 | 0.036 | 0.560 |
| 41661922 | 13 | rs7994783 | G | T | 0.302 | -0.009 | 0.001 | 1.70E-09 | 81 | -0.005 | 0.026 | 0.857 |
| 92015977 | 13 | rs8002779 | A | G | 0.603 | -0.007 | 0.001 | 3.10E-08 | 49 | -0.007 | 0.023 | 0.754 |
| 73358764 | 14 | rs8006178 | C | A | 0.228 | 0.009 | 0.002 | 2.10E-08 | 20 | 0.002 | 0.026 | 0.931 |
| 103987305 | 14 | rs8017780 | A | C | 0.213 | -0.010 | 0.002 | 1.70E-09 | 25 | 0.027 | 0.030 | 0.370 |
| 75822230 | 15 | rs8024244 | A | G | 0.251 | 0.010 | 0.002 | 2.80E-10 | 25 | -0.015 | 0.028 | 0.600 |
| 38367091 | 15 | rs8034033 | A | C | 0.708 | -0.012 | 0.001 | 2.90E-16 | 144 | -0.053 | 0.027 | 0.050 |
| 41903965 | 15 | rs8036643 | C | G | 0.646 | -0.010 | 0.001 | 2.00E-14 | 100 | 0.014 | 0.025 | 0.575 |
| 66992821 | 15 | rs8042545 | A | G | 0.242 | 0.013 | 0.002 | 8.90E-18 | 42 | -0.022 | 0.027 | 0.423 |
| 36951278 | 17 | rs8064547 | A | G | 0.520 | 0.009 | 0.001 | 1.40E-11 | 81 | 0.038 | 0.023 | 0.098 |
| 43257270 | 17 | rs8070437 | T | C | 0.704 | 0.013 | 0.001 | 3.10E-20 | 169 | 0.008 | 0.025 | 0.741 |
| 17407816 | 17 | rs8074074 | T | C | 0.755 | 0.010 | 0.002 | 1.80E-10 | 25 | 0.001 | 0.025 | 0.958 |
| 148650375 | 7 | rs822551 | G | A | 0.812 | 0.013 | 0.002 | 6.90E-14 | 42 | 0.066 | 0.031 | 0.031 |
| 53493387 | 12 | rs822688 | T | C | 0.124 | 0.015 | 0.002 | 2.60E-13 | 56 | -0.006 | 0.030 | 0.831 |
| 205723572 | 1 | rs823118 | T | C | 0.548 | -0.013 | 0.001 | 1.60E-24 | 169 | -0.010 | 0.023 | 0.676 |
| 6458319 | 7 | rs836511 | G | A | 0.199 | 0.011 | 0.002 | 2.70E-12 | 30 | 0.005 | 0.026 | 0.859 |
| 183997261 | 3 | rs843374 | T | A | 0.587 | -0.011 | 0.001 | 4.40E-17 | 121 | -0.008 | 0.023 | 0.741 |
| 88913273 | 2 | rs867529 | C | G | 0.279 | 0.010 | 0.001 | 2.00E-11 | 100 | 0.029 | 0.024 | 0.227 |
| 133431902 | 9 | rs878347 | C | T | 0.367 | -0.009 | 0.001 | 2.40E-10 | 81 | -0.055 | 0.023 | 0.016 |
| 135601194 | 8 | rs894360 | C | T | 0.366 | -0.019 | 0.001 | 1.10E-44 | 361 | -0.035 | 0.023 | 0.124 |
| 67599656 | 5 | rs9291926 | G | T | 0.532 | -0.013 | 0.001 | 1.00E-21 | 169 | -0.002 | 0.023 | 0.946 |
| 123990270 | 5 | rs9327336 | C | T | 0.343 | 0.009 | 0.001 | 4.60E-11 | 81 | 0.010 | 0.023 | 0.651 |
| 81050236 | 6 | rs9350850 | C | T | 0.079 | 0.022 | 0.002 | 1.00E-20 | 121 | 0.026 | 0.036 | 0.464 |
| 7231843 | 6 | rs9379084 | A | G | 0.116 | -0.015 | 0.002 | 2.30E-12 | 56 | 0.019 | 0.036 | 0.596 |
| 28012527 | 13 | rs9512696 | G | A | 0.662 | 0.010 | 0.001 | 3.60E-14 | 100 | -0.003 | 0.024 | 0.898 |
| 99571922 | 13 | rs9513510 | C | G | 0.699 | -0.010 | 0.001 | 5.20E-13 | 100 | 0.008 | 0.024 | 0.729 |
| 66205704 | 13 | rs9540493 | G | A | 0.545 | -0.010 | 0.001 | 1.90E-13 | 100 | -0.014 | 0.023 | 0.551 |
| 93993266 | 12 | rs9634212 | A | C | 0.221 | 0.023 | 0.002 | 1.30E-48 | 132 | 0.017 | 0.026 | 0.525 |
| 94187634 | 9 | rs968821 | C | G | 0.340 | -0.012 | 0.001 | 4.80E-18 | 144 | 0.014 | 0.026 | 0.577 |
| 23878279 | 14 | rs9788443 | C | T | 0.048 | 0.019 | 0.003 | 3.10E-10 | 40 | 0.040 | 0.094 | 0.673 |
| 77641008 | 5 | rs9800418 | C | T | 0.245 | 0.009 | 0.002 | 3.60E-09 | 20 | -0.056 | 0.026 | 0.032 |
| 56882326 | 18 | rs9951619 | G | T | 0.767 | 0.013 | 0.002 | 2.80E-17 | 42 | -0.022 | 0.024 | 0.366 |
| 33040095 | 18 | rs9960619 | T | C | 0.344 | 0.009 | 0.001 | 1.90E-11 | 81 | 0.001 | 0.024 | 0.972 |
| 135213286 | 4 | rs9985795 | C | T | 0.483 | -0.008 | 0.001 | 3.70E-09 | 64 | 0.018 | 0.023 | 0.437 |

BMR, basal metabolic rate; EAF, effect allele frequency; SNP, single nucleotide polymorphism; SE, standard error; PE, pulmonary embolism.
