# Supplementary material for: Genetically predicted basal metabolic rate and venous thromboembolism risk: a Mendelian randomization study
Source: Front Nutr. 2023 Dec 21;10:1263804. doi: 10.3389/fnut.2023.1263804 (PMC10768029; doi:10.3389/fnut.2023.1263804)
Supplement: Supplementary file 7 [file Table_7.DOCX]

Supplementary Table 7 Power calculation.

| Source for outcome dataset | Outcome | OR ≥ |
| --- | --- | --- |
| FinnGen | VTE | 1.160 |
|  | PE | 1.234 |
|  | DVT of lower extremities | 1.224 |

ORs were calculated by setting 80% of power. Specifically, we set alpha level at 0.05 and used the variance of BMR explained by its respective instrumental variables (3.5%) and study sample size.
