# Supplementary material for: Genetically predicted basal metabolic rate and venous thromboembolism risk: a Mendelian randomization study
Source: Front Nutr. 2023 Dec 21;10:1263804. doi: 10.3389/fnut.2023.1263804 (PMC10768029; doi:10.3389/fnut.2023.1263804)
Supplement: Supplementary file 9 [file Table_9.DOCX]

Supplementary Table 9 Leave-one-out sensitivity analysis for the association between BMR and PE.

| Instrumental variable | OR | 95% lower confidence interval | 95% upper confidence interval |
| --- | --- | --- | --- |
| All | 1.824 | 1.512 | 2.200 |
| rs10020631 | 1.820 | 1.509 | 2.196 |
| rs10058393 | 1.825 | 1.512 | 2.202 |
| rs1008158 | 1.818 | 1.507 | 2.193 |
| rs10172196 | 1.820 | 1.508 | 2.196 |
| rs10176567 | 1.829 | 1.516 | 2.206 |
| rs10188231 | 1.822 | 1.510 | 2.199 |
| rs10202845 | 1.830 | 1.517 | 2.208 |
| rs10236214 | 1.841 | 1.526 | 2.222 |
| rs10248298 | 1.815 | 1.504 | 2.189 |
| rs10260993 | 1.821 | 1.510 | 2.198 |
| rs10269774 | 1.793 | 1.486 | 2.164 |
| rs10401784 | 1.808 | 1.500 | 2.180 |
| rs10404726 | 1.817 | 1.506 | 2.192 |
| rs1045475 | 1.831 | 1.517 | 2.210 |
| rs10457469 | 1.825 | 1.512 | 2.202 |
| rs1047891 | 1.807 | 1.498 | 2.180 |
| rs10511111 | 1.825 | 1.513 | 2.203 |
| rs1064213 | 1.804 | 1.497 | 2.173 |
| rs10740991 | 1.818 | 1.507 | 2.193 |
| rs10748128 | 1.822 | 1.510 | 2.199 |
| rs10773172 | 1.833 | 1.519 | 2.212 |
| rs10775348 | 1.820 | 1.508 | 2.196 |
| rs10775406 | 1.823 | 1.511 | 2.200 |
| rs10777860 | 1.824 | 1.512 | 2.201 |
| rs10803955 | 1.819 | 1.507 | 2.194 |
| rs10822373 | 1.831 | 1.518 | 2.209 |
| rs10843397 | 1.824 | 1.512 | 2.201 |
| rs10870597 | 1.836 | 1.522 | 2.213 |
| rs10886477 | 1.828 | 1.515 | 2.205 |
| rs10887571 | 1.816 | 1.506 | 2.191 |
| rs10914462 | 1.823 | 1.511 | 2.200 |
| rs10938397 | 1.822 | 1.509 | 2.198 |
| rs10939792 | 1.825 | 1.512 | 2.202 |
| rs10979612 | 1.838 | 1.524 | 2.217 |
| rs10995366 | 1.834 | 1.521 | 2.212 |
| rs11014285 | 1.831 | 1.517 | 2.209 |
| rs11018104 | 1.822 | 1.510 | 2.198 |
| rs11030112 | 1.813 | 1.502 | 2.188 |
| rs11049684 | 1.824 | 1.512 | 2.202 |
| rs11065015 | 1.833 | 1.519 | 2.212 |
| rs11065979 | 1.832 | 1.519 | 2.211 |
| rs1108548 | 1.826 | 1.513 | 2.204 |
| rs11097755 | 1.828 | 1.515 | 2.205 |
| rs111365325 | 1.805 | 1.497 | 2.175 |
| rs11142700 | 1.829 | 1.516 | 2.207 |
| rs11153171 | 1.828 | 1.515 | 2.206 |
| rs111598585 | 1.817 | 1.507 | 2.190 |
| rs11160601 | 1.827 | 1.514 | 2.205 |
| rs111640872 | 1.811 | 1.501 | 2.185 |
| rs11175890 | 1.826 | 1.513 | 2.203 |
| rs11196652 | 1.828 | 1.515 | 2.206 |
| rs11205354 | 1.823 | 1.511 | 2.199 |
| rs112069922 | 1.823 | 1.511 | 2.200 |
| rs11217843 | 1.805 | 1.498 | 2.175 |
| rs11243202 | 1.827 | 1.513 | 2.205 |
| rs11245450 | 1.818 | 1.507 | 2.194 |
| rs112544217 | 1.827 | 1.514 | 2.204 |
| rs112560164 | 1.822 | 1.510 | 2.199 |
| rs112685832 | 1.830 | 1.517 | 2.208 |
| rs112758380 | 1.818 | 1.506 | 2.193 |
| rs112875651 | 1.827 | 1.514 | 2.205 |
| rs112957890 | 1.825 | 1.513 | 2.202 |
| rs113619763 | 1.824 | 1.511 | 2.201 |
| rs114056237 | 1.827 | 1.515 | 2.205 |
| rs114177791 | 1.827 | 1.514 | 2.204 |
| rs115179432 | 1.822 | 1.510 | 2.199 |
| rs11525873 | 1.830 | 1.516 | 2.208 |
| rs11545482 | 1.825 | 1.513 | 2.202 |
| rs11578046 | 1.819 | 1.507 | 2.195 |
| rs115946508 | 1.822 | 1.510 | 2.198 |
| rs11611651 | 1.826 | 1.513 | 2.203 |
| rs11616283 | 1.822 | 1.510 | 2.199 |
| rs116165844 | 1.824 | 1.512 | 2.201 |
| rs116337081 | 1.824 | 1.512 | 2.201 |
| rs11663903 | 1.820 | 1.509 | 2.196 |
| rs11684531 | 1.824 | 1.512 | 2.201 |
| rs11688707 | 1.829 | 1.516 | 2.207 |
| rs117034105 | 1.823 | 1.511 | 2.200 |
| rs11709402 | 1.821 | 1.509 | 2.197 |
| rs11739036 | 1.817 | 1.506 | 2.192 |
| rs117451679 | 1.828 | 1.515 | 2.206 |
| rs117543413 | 1.824 | 1.511 | 2.201 |
| rs11756675 | 1.832 | 1.519 | 2.209 |
| rs1176314 | 1.823 | 1.511 | 2.200 |
| rs11777007 | 1.815 | 1.505 | 2.189 |
| rs11785562 | 1.819 | 1.507 | 2.194 |
| rs11794152 | 1.817 | 1.506 | 2.193 |
| rs1179905 | 1.822 | 1.510 | 2.199 |
| rs118173451 | 1.828 | 1.515 | 2.206 |
| rs11880992 | 1.819 | 1.507 | 2.195 |
| rs11997525 | 1.830 | 1.517 | 2.208 |
| rs12047401 | 1.840 | 1.528 | 2.216 |
| rs1205593 | 1.812 | 1.502 | 2.186 |
| rs12083887 | 1.819 | 1.508 | 2.194 |
| rs12123505 | 1.834 | 1.521 | 2.211 |
| rs12129705 | 1.818 | 1.507 | 2.193 |
| rs12140153 | 1.817 | 1.506 | 2.192 |
| rs12156265 | 1.829 | 1.516 | 2.206 |
| rs12188627 | 1.828 | 1.515 | 2.206 |
| rs12200061 | 1.830 | 1.517 | 2.207 |
| rs12209223 | 1.826 | 1.513 | 2.203 |
| rs12213070 | 1.810 | 1.502 | 2.182 |
| rs12216497 | 1.818 | 1.507 | 2.194 |
| rs1228024 | 1.816 | 1.505 | 2.190 |
| rs12294689 | 1.820 | 1.509 | 2.195 |
| rs12364470 | 1.819 | 1.507 | 2.194 |
| rs12427047 | 1.839 | 1.525 | 2.217 |
| rs12553221 | 1.831 | 1.518 | 2.208 |
| rs1260326 | 1.836 | 1.521 | 2.215 |
| rs12657771 | 1.820 | 1.508 | 2.196 |
| rs12694042 | 1.827 | 1.514 | 2.204 |
| rs12713004 | 1.826 | 1.513 | 2.203 |
| rs12731187 | 1.830 | 1.517 | 2.207 |
| rs12731454 | 1.816 | 1.506 | 2.191 |
| rs12763284 | 1.815 | 1.504 | 2.189 |
| rs12764498 | 1.809 | 1.501 | 2.181 |
| rs12820906 | 1.835 | 1.522 | 2.213 |
| rs12879423 | 1.835 | 1.521 | 2.214 |
| rs12888955 | 1.824 | 1.512 | 2.201 |
| rs12889702 | 1.834 | 1.520 | 2.212 |
| rs12907384 | 1.822 | 1.510 | 2.199 |
| rs12908182 | 1.827 | 1.514 | 2.204 |
| rs12926311 | 1.825 | 1.513 | 2.202 |
| rs12934835 | 1.822 | 1.510 | 2.199 |
| rs1296328 | 1.827 | 1.514 | 2.205 |
| rs13043303 | 1.831 | 1.518 | 2.210 |
| rs13059004 | 1.824 | 1.512 | 2.201 |
| rs13085472 | 1.816 | 1.506 | 2.191 |
| rs13125807 | 1.817 | 1.506 | 2.193 |
| rs13225455 | 1.821 | 1.510 | 2.198 |
| rs13244614 | 1.816 | 1.506 | 2.191 |
| rs13299559 | 1.818 | 1.506 | 2.193 |
| rs13430869 | 1.828 | 1.515 | 2.206 |
| rs1351394 | 1.824 | 1.511 | 2.203 |
| rs1374370 | 1.827 | 1.514 | 2.204 |
| rs1407031 | 1.830 | 1.517 | 2.207 |
| rs1412234 | 1.827 | 1.514 | 2.205 |
| rs1418433 | 1.834 | 1.521 | 2.212 |
| rs1421035 | 1.829 | 1.516 | 2.206 |
| rs143384 | 1.810 | 1.498 | 2.187 |
| rs1443536 | 1.825 | 1.512 | 2.202 |
| rs1447498 | 1.825 | 1.513 | 2.202 |
| rs145391487 | 1.836 | 1.523 | 2.213 |
| rs146851424 | 1.814 | 1.503 | 2.189 |
| rs147110934 | 1.823 | 1.511 | 2.200 |
| rs147233090 | 1.822 | 1.510 | 2.199 |
| rs148662000 | 1.829 | 1.517 | 2.207 |
| rs1521624 | 1.821 | 1.509 | 2.197 |
| rs1524445 | 1.831 | 1.518 | 2.209 |
| rs1542224 | 1.821 | 1.509 | 2.197 |
| rs1553065 | 1.824 | 1.512 | 2.201 |
| rs1573891 | 1.834 | 1.520 | 2.212 |
| rs1582931 | 1.839 | 1.524 | 2.219 |
| rs1640779 | 1.821 | 1.509 | 2.198 |
| rs1657222 | 1.837 | 1.524 | 2.214 |
| rs16916881 | 1.826 | 1.513 | 2.203 |
| rs16942324 | 1.820 | 1.508 | 2.195 |
| rs16996657 | 1.819 | 1.508 | 2.195 |
| rs17010961 | 1.815 | 1.505 | 2.190 |
| rs17024393 | 1.817 | 1.505 | 2.192 |
| rs17042011 | 1.828 | 1.515 | 2.205 |
| rs17069647 | 1.821 | 1.509 | 2.197 |
| rs17115481 | 1.831 | 1.518 | 2.209 |
| rs17157112 | 1.818 | 1.507 | 2.193 |
| rs1717726 | 1.827 | 1.514 | 2.204 |
| rs17197114 | 1.821 | 1.509 | 2.197 |
| rs17246129 | 1.821 | 1.509 | 2.197 |
| rs17363646 | 1.822 | 1.510 | 2.199 |
| rs17400325 | 1.835 | 1.523 | 2.211 |
| rs17443541 | 1.829 | 1.516 | 2.206 |
| rs17454077 | 1.818 | 1.507 | 2.192 |
| rs17491275 | 1.823 | 1.511 | 2.201 |
| rs1780673 | 1.832 | 1.518 | 2.210 |
| rs17828687 | 1.823 | 1.511 | 2.200 |
| rs1813212 | 1.825 | 1.512 | 2.202 |
| rs1837367 | 1.815 | 1.505 | 2.189 |
| rs1846221 | 1.820 | 1.509 | 2.196 |
| rs1910466 | 1.822 | 1.510 | 2.198 |
| rs1923766 | 1.824 | 1.512 | 2.201 |
| rs1924936 | 1.806 | 1.498 | 2.178 |
| rs1927635 | 1.829 | 1.516 | 2.207 |
| rs1941697 | 1.821 | 1.509 | 2.198 |
| rs1966818 | 1.829 | 1.516 | 2.206 |
| rs1984119 | 1.829 | 1.515 | 2.207 |
| rs2005172 | 1.817 | 1.505 | 2.193 |
| rs2016469 | 1.827 | 1.514 | 2.204 |
| rs2034923 | 1.817 | 1.506 | 2.192 |
| rs2071519 | 1.815 | 1.505 | 2.190 |
| rs2077218 | 1.820 | 1.509 | 2.196 |
| rs2093147 | 1.826 | 1.513 | 2.203 |
| rs2101975 | 1.828 | 1.515 | 2.206 |
| rs2102278 | 1.821 | 1.509 | 2.197 |
| rs2122823 | 1.827 | 1.514 | 2.204 |
| rs212526 | 1.830 | 1.517 | 2.208 |
| rs2140046 | 1.826 | 1.513 | 2.203 |
| rs2197563 | 1.828 | 1.515 | 2.206 |
| rs2225226 | 1.832 | 1.518 | 2.213 |
| rs2229840 | 1.819 | 1.507 | 2.196 |
| rs223942 | 1.824 | 1.512 | 2.201 |
| rs224048 | 1.822 | 1.510 | 2.198 |
| rs225111 | 1.822 | 1.510 | 2.198 |
| rs2252720 | 1.830 | 1.516 | 2.208 |
| rs2270894 | 1.831 | 1.517 | 2.210 |
| rs2276190 | 1.809 | 1.501 | 2.181 |
| rs2281175 | 1.831 | 1.517 | 2.209 |
| rs2287214 | 1.828 | 1.516 | 2.206 |
| rs2293176 | 1.818 | 1.507 | 2.193 |
| rs2293888 | 1.835 | 1.521 | 2.215 |
| rs2296316 | 1.824 | 1.511 | 2.201 |
| rs2303792 | 1.822 | 1.510 | 2.198 |
| rs2307111 | 1.819 | 1.507 | 2.195 |
| rs236587 | 1.813 | 1.504 | 2.186 |
| rs2439823 | 1.818 | 1.507 | 2.193 |
| rs244711 | 1.824 | 1.511 | 2.201 |
| rs247008 | 1.832 | 1.519 | 2.211 |
| rs2521349 | 1.822 | 1.510 | 2.198 |
| rs252938 | 1.822 | 1.510 | 2.198 |
| rs2533879 | 1.806 | 1.497 | 2.180 |
| rs254963 | 1.826 | 1.513 | 2.203 |
| rs2568958 | 1.825 | 1.513 | 2.203 |
| rs2569993 | 1.826 | 1.514 | 2.204 |
| rs2578246 | 1.820 | 1.508 | 2.195 |
| rs2611732 | 1.834 | 1.521 | 2.211 |
| rs2615075 | 1.817 | 1.506 | 2.193 |
| rs2627692 | 1.826 | 1.514 | 2.204 |
| rs2650965 | 1.824 | 1.512 | 2.202 |
| rs2678204 | 1.821 | 1.509 | 2.198 |
| rs2699433 | 1.822 | 1.510 | 2.199 |
| rs2744956 | 1.797 | 1.487 | 2.170 |
| rs2756895 | 1.818 | 1.507 | 2.194 |
| rs2772435 | 1.807 | 1.501 | 2.175 |
| rs2789366 | 1.819 | 1.508 | 2.195 |
| rs281385 | 1.828 | 1.515 | 2.206 |
| rs284315 | 1.820 | 1.509 | 2.196 |
| rs284662 | 1.827 | 1.514 | 2.204 |
| rs28605759 | 1.814 | 1.504 | 2.188 |
| rs28620532 | 1.823 | 1.510 | 2.200 |
| rs2866719 | 1.820 | 1.509 | 2.197 |
| rs2897968 | 1.821 | 1.509 | 2.198 |
| rs2900208 | 1.826 | 1.513 | 2.204 |
| rs2952615 | 1.834 | 1.521 | 2.213 |
| rs29946 | 1.815 | 1.505 | 2.188 |
| rs310796 | 1.831 | 1.518 | 2.209 |
| rs3110496 | 1.835 | 1.522 | 2.213 |
| rs3116201 | 1.826 | 1.514 | 2.204 |
| rs31211 | 1.832 | 1.518 | 2.210 |
| rs3212260 | 1.819 | 1.507 | 2.194 |
| rs34017457 | 1.825 | 1.512 | 2.202 |
| rs34055910 | 1.827 | 1.514 | 2.205 |
| rs34147411 | 1.832 | 1.519 | 2.210 |
| rs34227797 | 1.821 | 1.509 | 2.197 |
| rs34268501 | 1.824 | 1.511 | 2.200 |
| rs34373881 | 1.822 | 1.510 | 2.199 |
| rs34517439 | 1.800 | 1.491 | 2.173 |
| rs34693680 | 1.824 | 1.511 | 2.201 |
| rs34776209 | 1.827 | 1.514 | 2.205 |
| rs34825238 | 1.821 | 1.509 | 2.196 |
| rs34848742 | 1.826 | 1.513 | 2.203 |
| rs34879158 | 1.821 | 1.508 | 2.198 |
| rs35050648 | 1.828 | 1.515 | 2.205 |
| rs35251247 | 1.815 | 1.505 | 2.190 |
| rs35467921 | 1.817 | 1.505 | 2.194 |
| rs35506085 | 1.823 | 1.511 | 2.201 |
| rs35665085 | 1.820 | 1.508 | 2.196 |
| rs35679149 | 1.828 | 1.515 | 2.205 |
| rs357486 | 1.836 | 1.523 | 2.215 |
| rs35874463 | 1.828 | 1.515 | 2.205 |
| rs36000545 | 1.839 | 1.524 | 2.219 |
| rs36012032 | 1.813 | 1.503 | 2.186 |
| rs36100359 | 1.827 | 1.514 | 2.204 |
| rs365352 | 1.823 | 1.511 | 2.200 |
| rs36695 | 1.833 | 1.520 | 2.210 |
| rs3730071 | 1.821 | 1.509 | 2.196 |
| rs3740591 | 1.823 | 1.511 | 2.200 |
| rs3744806 | 1.833 | 1.520 | 2.211 |
| rs3759094 | 1.835 | 1.521 | 2.213 |
| rs3765351 | 1.821 | 1.509 | 2.197 |
| rs3778934 | 1.825 | 1.512 | 2.202 |
| rs3800963 | 1.827 | 1.514 | 2.204 |
| rs3810291 | 1.828 | 1.515 | 2.207 |
| rs3812550 | 1.827 | 1.514 | 2.205 |
| rs3822742 | 1.831 | 1.517 | 2.209 |
| rs3845344 | 1.824 | 1.512 | 2.201 |
| rs3853252 | 1.830 | 1.517 | 2.209 |
| rs3853474 | 1.824 | 1.512 | 2.201 |
| rs3925 | 1.825 | 1.513 | 2.202 |
| rs41271299 | 1.832 | 1.518 | 2.210 |
| rs41311445 | 1.830 | 1.516 | 2.209 |
| rs4132132 | 1.832 | 1.519 | 2.210 |
| rs41458449 | 1.834 | 1.521 | 2.211 |
| rs4240326 | 1.822 | 1.509 | 2.200 |
| rs4253755 | 1.824 | 1.512 | 2.201 |
| rs4268495 | 1.818 | 1.507 | 2.194 |
| rs4282339 | 1.816 | 1.505 | 2.191 |
| rs4311660 | 1.821 | 1.509 | 2.197 |
| rs4369779 | 1.817 | 1.505 | 2.194 |
| rs4525525 | 1.823 | 1.511 | 2.200 |
| rs4556997 | 1.837 | 1.524 | 2.215 |
| rs4567604 | 1.827 | 1.514 | 2.204 |
| rs4619406 | 1.817 | 1.506 | 2.192 |
| rs4635681 | 1.836 | 1.522 | 2.214 |
| rs4648626 | 1.827 | 1.514 | 2.204 |
| rs4653016 | 1.831 | 1.518 | 2.210 |
| rs4740292 | 1.821 | 1.509 | 2.196 |
| rs475390 | 1.818 | 1.506 | 2.194 |
| rs4776970 | 1.807 | 1.498 | 2.178 |
| rs4782286 | 1.834 | 1.521 | 2.212 |
| rs4819021 | 1.817 | 1.506 | 2.192 |
| rs485554 | 1.817 | 1.506 | 2.193 |
| rs4858940 | 1.829 | 1.516 | 2.207 |
| rs4865465 | 1.828 | 1.515 | 2.205 |
| rs4865956 | 1.829 | 1.516 | 2.207 |
| rs4883723 | 1.809 | 1.500 | 2.181 |
| rs4899012 | 1.830 | 1.516 | 2.209 |
| rs4912905 | 1.828 | 1.515 | 2.206 |
| rs4946936 | 1.839 | 1.524 | 2.220 |
| rs4968799 | 1.827 | 1.514 | 2.205 |
| rs4974072 | 1.839 | 1.525 | 2.217 |
| rs4980826 | 1.821 | 1.509 | 2.197 |
| rs5017213 | 1.822 | 1.510 | 2.199 |
| rs5019542 | 1.827 | 1.514 | 2.205 |
| rs505575 | 1.825 | 1.512 | 2.202 |
| rs508347 | 1.811 | 1.501 | 2.184 |
| rs522468 | 1.817 | 1.506 | 2.191 |
| rs532499 | 1.825 | 1.512 | 2.202 |
| rs543874 | 1.837 | 1.521 | 2.217 |
| rs55674305 | 1.820 | 1.508 | 2.196 |
| rs55726687 | 1.829 | 1.515 | 2.207 |
| rs55758152 | 1.829 | 1.516 | 2.207 |
| rs55831773 | 1.819 | 1.507 | 2.195 |
| rs55854145 | 1.824 | 1.512 | 2.201 |
| rs56094641 | 1.796 | 1.485 | 2.171 |
| rs56130943 | 1.829 | 1.516 | 2.207 |
| rs56383938 | 1.830 | 1.516 | 2.207 |
| rs57126421 | 1.827 | 1.514 | 2.204 |
| rs573455 | 1.826 | 1.513 | 2.203 |
| rs5752989 | 1.830 | 1.516 | 2.208 |
| rs57636386 | 1.818 | 1.507 | 2.193 |
| rs5771118 | 1.825 | 1.512 | 2.202 |
| rs578366 | 1.828 | 1.515 | 2.206 |
| rs58584712 | 1.820 | 1.509 | 2.196 |
| rs599004 | 1.822 | 1.510 | 2.198 |
| rs59985551 | 1.808 | 1.498 | 2.181 |
| rs60077625 | 1.809 | 1.500 | 2.182 |
| rs6026578 | 1.831 | 1.518 | 2.209 |
| rs6130953 | 1.824 | 1.512 | 2.201 |
| rs6136938 | 1.833 | 1.519 | 2.211 |
| rs6142059 | 1.829 | 1.516 | 2.207 |
| rs61628776 | 1.823 | 1.510 | 2.199 |
| rs61729527 | 1.823 | 1.511 | 2.200 |
| rs61828917 | 1.830 | 1.518 | 2.208 |
| rs61869763 | 1.811 | 1.502 | 2.184 |
| rs61992671 | 1.836 | 1.522 | 2.214 |
| rs62246311 | 1.822 | 1.510 | 2.199 |
| rs6235 | 1.819 | 1.507 | 2.195 |
| rs62372052 | 1.829 | 1.515 | 2.208 |
| rs62396185 | 1.821 | 1.509 | 2.199 |
| rs62439025 | 1.825 | 1.512 | 2.202 |
| rs62460522 | 1.822 | 1.510 | 2.199 |
| rs62466118 | 1.824 | 1.512 | 2.201 |
| rs62515437 | 1.821 | 1.508 | 2.198 |
| rs62621197 | 1.814 | 1.504 | 2.188 |
| rs62621400 | 1.817 | 1.506 | 2.193 |
| rs62621812 | 1.838 | 1.524 | 2.218 |
| rs6437277 | 1.817 | 1.506 | 2.192 |
| rs6493780 | 1.825 | 1.512 | 2.202 |
| rs6500249 | 1.826 | 1.513 | 2.203 |
| rs655598 | 1.823 | 1.511 | 2.200 |
| rs6563808 | 1.821 | 1.509 | 2.197 |
| rs6567160 | 1.825 | 1.510 | 2.205 |
| rs6570509 | 1.828 | 1.515 | 2.206 |
| rs6575340 | 1.826 | 1.513 | 2.203 |
| rs6585827 | 1.825 | 1.512 | 2.202 |
| rs667515 | 1.821 | 1.509 | 2.198 |
| rs6675441 | 1.816 | 1.506 | 2.191 |
| rs6681795 | 1.803 | 1.496 | 2.174 |
| rs67141907 | 1.819 | 1.508 | 2.194 |
| rs6743060 | 1.820 | 1.507 | 2.199 |
| rs67551338 | 1.832 | 1.518 | 2.210 |
| rs6762578 | 1.817 | 1.506 | 2.193 |
| rs6762851 | 1.821 | 1.509 | 2.197 |
| rs6777784 | 1.824 | 1.512 | 2.201 |
| rs6779752 | 1.821 | 1.509 | 2.197 |
| rs6792892 | 1.830 | 1.516 | 2.209 |
| rs68106312 | 1.829 | 1.516 | 2.207 |
| rs6874142 | 1.826 | 1.513 | 2.204 |
| rs6951489 | 1.822 | 1.510 | 2.199 |
| rs695922 | 1.812 | 1.503 | 2.184 |
| rs6984820 | 1.826 | 1.513 | 2.204 |
| rs700768 | 1.815 | 1.505 | 2.189 |
| rs7033487 | 1.830 | 1.516 | 2.209 |
| rs703593 | 1.826 | 1.513 | 2.204 |
| rs7109581 | 1.821 | 1.509 | 2.197 |
| rs7111235 | 1.823 | 1.511 | 2.200 |
| rs7129320 | 1.835 | 1.521 | 2.214 |
| rs7132908 | 1.812 | 1.501 | 2.187 |
| rs7134283 | 1.822 | 1.510 | 2.198 |
| rs71385734 | 1.841 | 1.526 | 2.221 |
| rs7141420 | 1.811 | 1.501 | 2.185 |
| rs71423263 | 1.829 | 1.516 | 2.207 |
| rs7188009 | 1.832 | 1.519 | 2.210 |
| rs7223535 | 1.809 | 1.499 | 2.184 |
| rs7229520 | 1.821 | 1.509 | 2.198 |
| rs723149 | 1.836 | 1.522 | 2.215 |
| rs724016 | 1.836 | 1.520 | 2.218 |
| rs7245985 | 1.820 | 1.508 | 2.196 |
| rs726547 | 1.825 | 1.512 | 2.202 |
| rs72656010 | 1.816 | 1.504 | 2.192 |
| rs72703409 | 1.821 | 1.509 | 2.197 |
| rs72722756 | 1.818 | 1.507 | 2.193 |
| rs72801843 | 1.829 | 1.515 | 2.207 |
| rs72885917 | 1.819 | 1.507 | 2.196 |
| rs72892910 | 1.827 | 1.513 | 2.205 |
| rs73004967 | 1.825 | 1.512 | 2.202 |
| rs7301341 | 1.828 | 1.515 | 2.206 |
| rs73013411 | 1.822 | 1.510 | 2.199 |
| rs73052033 | 1.830 | 1.517 | 2.208 |
| rs73093103 | 1.821 | 1.509 | 2.197 |
| rs7312646 | 1.831 | 1.518 | 2.209 |
| rs73175572 | 1.833 | 1.519 | 2.212 |
| rs73189390 | 1.827 | 1.514 | 2.204 |
| rs7321045 | 1.822 | 1.510 | 2.199 |
| rs73213484 | 1.814 | 1.504 | 2.189 |
| rs73601548 | 1.819 | 1.508 | 2.195 |
| rs73619441 | 1.817 | 1.506 | 2.191 |
| rs74032128 | 1.819 | 1.508 | 2.194 |
| rs74048171 | 1.824 | 1.511 | 2.201 |
| rs744205 | 1.827 | 1.514 | 2.205 |
| rs74494415 | 1.818 | 1.506 | 2.194 |
| rs7513326 | 1.822 | 1.510 | 2.198 |
| rs7519259 | 1.815 | 1.505 | 2.188 |
| rs752070 | 1.825 | 1.512 | 2.202 |
| rs754635 | 1.831 | 1.518 | 2.208 |
| rs75581912 | 1.828 | 1.515 | 2.206 |
| rs757608 | 1.823 | 1.510 | 2.200 |
| rs757833 | 1.832 | 1.519 | 2.209 |
| rs76067562 | 1.836 | 1.523 | 2.213 |
| rs76116290 | 1.828 | 1.515 | 2.205 |
| rs7612882 | 1.828 | 1.516 | 2.206 |
| rs7613368 | 1.832 | 1.518 | 2.209 |
| rs7619139 | 1.827 | 1.514 | 2.205 |
| rs76307059 | 1.820 | 1.508 | 2.195 |
| rs76513770 | 1.829 | 1.515 | 2.207 |
| rs7671110 | 1.831 | 1.517 | 2.210 |
| rs76798800 | 1.794 | 1.488 | 2.164 |
| rs7683836 | 1.825 | 1.512 | 2.202 |
| rs76895963 | 1.812 | 1.500 | 2.189 |
| rs77093479 | 1.838 | 1.525 | 2.214 |
| rs77165542 | 1.836 | 1.521 | 2.215 |
| rs7728690 | 1.820 | 1.509 | 2.197 |
| rs7731023 | 1.816 | 1.506 | 2.191 |
| rs7740107 | 1.844 | 1.527 | 2.226 |
| rs7755185 | 1.828 | 1.515 | 2.205 |
| rs7780752 | 1.828 | 1.515 | 2.206 |
| rs77848106 | 1.822 | 1.510 | 2.199 |
| rs78149371 | 1.820 | 1.509 | 2.196 |
| rs7815955 | 1.826 | 1.513 | 2.203 |
| rs78287937 | 1.826 | 1.514 | 2.203 |
| rs78378222 | 1.831 | 1.517 | 2.210 |
| rs7842996 | 1.832 | 1.518 | 2.211 |
| rs7912286 | 1.832 | 1.519 | 2.210 |
| rs7927350 | 1.823 | 1.511 | 2.200 |
| rs7933085 | 1.826 | 1.513 | 2.203 |
| rs7952436 | 1.814 | 1.503 | 2.188 |
| rs7969505 | 1.822 | 1.510 | 2.198 |
| rs7994783 | 1.824 | 1.512 | 2.201 |
| rs8002779 | 1.823 | 1.511 | 2.200 |
| rs8006178 | 1.824 | 1.512 | 2.201 |
| rs8017780 | 1.829 | 1.516 | 2.206 |
| rs8024244 | 1.827 | 1.514 | 2.205 |
| rs8034033 | 1.814 | 1.504 | 2.187 |
| rs8036643 | 1.829 | 1.516 | 2.206 |
| rs8042545 | 1.831 | 1.518 | 2.209 |
| rs8064547 | 1.816 | 1.506 | 2.191 |
| rs8070437 | 1.824 | 1.511 | 2.201 |
| rs8074074 | 1.825 | 1.512 | 2.202 |
| rs822551 | 1.813 | 1.503 | 2.186 |
| rs822688 | 1.827 | 1.514 | 2.205 |
| rs823118 | 1.823 | 1.511 | 2.200 |
| rs836511 | 1.824 | 1.512 | 2.201 |
| rs843374 | 1.824 | 1.511 | 2.201 |
| rs867529 | 1.818 | 1.507 | 2.194 |
| rs878347 | 1.813 | 1.503 | 2.185 |
| rs894360 | 1.812 | 1.501 | 2.187 |
| rs9291926 | 1.826 | 1.513 | 2.203 |
| rs9327336 | 1.823 | 1.510 | 2.199 |
| rs9350850 | 1.821 | 1.509 | 2.197 |
| rs9379084 | 1.828 | 1.515 | 2.206 |
| rs9512696 | 1.826 | 1.513 | 2.204 |
| rs9513510 | 1.827 | 1.514 | 2.205 |
| rs9540493 | 1.822 | 1.510 | 2.198 |
| rs9634212 | 1.822 | 1.510 | 2.200 |
| rs968821 | 1.829 | 1.516 | 2.207 |
| rs9788443 | 1.823 | 1.511 | 2.200 |
| rs9800418 | 1.835 | 1.522 | 2.212 |
| rs9951619 | 1.833 | 1.519 | 2.212 |
| rs9960619 | 1.825 | 1.512 | 2.202 |
| rs9985795 | 1.828 | 1.516 | 2.206 |

OR, odds ratio.
