# Supplementary material for: Genetically predicted basal metabolic rate and venous thromboembolism risk: a Mendelian randomization study
Source: Front Nutr. 2023 Dec 21;10:1263804. doi: 10.3389/fnut.2023.1263804 (PMC10768029; doi:10.3389/fnut.2023.1263804)
Supplement: Supplementary file 11 [file Table_11.DOCX]

Supplementary Table 11 Characteristics of the instrumental variables for VTE and its subtypes and their relationship with BMR.

| Exposure | Position | Chr | SNP | EA | OA | EAF | Exposure effect |  |  | F-statistic | Outcome effect |  |  |
| --- | --- | --- | --- | --- | --- | --- | --- | --- | --- | --- | --- | --- | --- |
|  |  |  |  |  |  |  | Effect size (β) | SE | *P* |  | Effect size (β) | SE | *P* |
| VTE | 104240958 | 12 | rs117716477 | A | C | 0.017 | 0.486 | 0.066 | 1.76E-13 | 54 | 0.005 | 0.006 | 0.33 |
|  | 71213386 | 10 | rs13377102 | A | T | 0.109 | -0.184 | 0.026 | 9.22E-13 | 50 | 0.002 | 0.002 | 0.34 |
|  | 169467654 | 1 | rs1894692 | A | G | 0.980 | -1.177 | 0.063 | 4.27E-79 | 349 | -0.009 | 0.005 | 0.04 |
|  | 155525276 | 4 | rs2066865 | A | G | 0.302 | 0.217 | 0.017 | 6.92E-36 | 163 | -0.003 | 0.002 | 0.08 |
|  | 10729097 | 19 | rs2885055 | A | G | 0.822 | 0.138 | 0.021 | 3.30E-11 | 43 | -0.013 | 0.002 | 1.50E-16 |
|  | 187206249 | 4 | rs3756011 | A | C | 0.427 | 0.204 | 0.016 | 2.89E-37 | 163 | 0.005 | 0.001 | 4.50E-04 |
|  | 136145240 | 9 | rs495203 | T | C | 0.436 | 0.250 | 0.016 | 4.05E-55 | 244 | -0.004 | 0.001 | 0.01 |
|  | 46745003 | 11 | rs5896 | T | C | 0.222 | 0.113 | 0.020 | 2.44E-08 | 32 | 0.002 | 0.002 | 0.2 |
|  | 33794378 | 20 | rs6060308 | A | G | 0.318 | 0.114 | 0.017 | 2.17E-11 | 45 | 0.017 | 0.001 | 1.10E-30 |
|  | 187277666 | 4 | rs62350309 | G | A | 0.103 | -0.164 | 0.026 | 4.71E-10 | 40 | -0.002 | 0.003 | 0.55 |
|  | 135877287 | 9 | rs628094 | A | G | 0.683 | 0.104 | 0.017 | 1.13E-09 | 37 | 0.000 | 0.001 | 0.8 |
|  | 46596670 | 11 | rs78807356 | T | G | 0.007 | 0.903 | 0.105 | 8.05E-18 | 74 | 0.004 | 0.004 | 0.29 |
|  | 31349819 | 6 | rs9266721 | A | C | 0.602 | -0.090 | 0.016 | 2.89E-08 | 32 | 0.002 | 0.001 | 0.17 |
| PE | 104240958 | 12 | rs117716477 | A | C | 0.017 | 0.590 | 0.097 | 1.04E-09 | 37 | 0.005 | 0.006 | 0.3300 |
|  | 169467654 | 1 | rs1894692 | A | G | 0.980 | -0.752 | 0.087 | 6.48E-18 | 75 | -0.009 | 0.005 | 0.0410 |
|  | 10738639 | 19 | rs3087969 | T | C | 0.824 | 0.177 | 0.030 | 3.55E-09 | 35 | -0.013 | 0.002 | 9.10E-17 |
|  | 187206249 | 4 | rs3756011 | A | C | 0.427 | 0.257 | 0.023 | 6.06E-29 | 125 | 0.005 | 0.001 | 0.0004 |
|  | 136142355 | 9 | rs643434 | A | G | 0.457 | 0.205 | 0.023 | 2.55E-19 | 79 | -0.003 | 0.001 | 0.0170 |
|  | 155520930 | 4 | rs7659024 | A | G | 0.302 | 0.286 | 0.025 | 2.53E-30 | 131 | -0.003 | 0.002 | 0.0760 |
| DVT of lower extremities | 46730639 | 11 | rs11602537 | G | C | 0.222 | 0.168 | 0.028 | 2.69E-09 | 36 | 0.003 | 0.002 | 0.1700 |
|  | 104240958 | 12 | rs117716477 | A | C | 0.016 | 0.542 | 0.092 | 3.49E-09 | 35 | 0.005 | 0.006 | 0.3300 |
|  | 155514879 | 4 | rs13109457 | A | G | 0.314 | 0.191 | 0.024 | 1.06E-15 | 63 | -0.003 | 0.001 | 0.0350 |
|  | 71213386 | 10 | rs13377102 | A | T | 0.109 | -0.201 | 0.036 | 1.71E-08 | 31 | 0.002 | 0.002 | 0.3400 |
|  | 169467654 | 1 | rs1894692 | A | G | 0.980 | -1.746 | 0.091 | 1.86E-82 | 368 | -0.009 | 0.005 | 0.0410 |
|  | 187206249 | 4 | rs3756011 | A | C | 0.427 | 0.213 | 0.022 | 1.21E-21 | 94 | 0.005 | 0.001 | 0.0004 |
|  | 136149500 | 9 | rs529565 | C | T | 0.434 | 0.324 | 0.022 | 1.08E-47 | 217 | -0.004 | 0.001 | 0.0084 |
|  | 33794378 | 20 | rs6060308 | A | G | 0.318 | 0.158 | 0.024 | 2.89E-11 | 43 | 0.017 | 0.001 | 1.10E-30 |
|  | 46596670 | 11 | rs78807356 | T | G | 0.007 | 1.173 | 0.150 | 5.59E-15 | 61 | 0.004 | 0.004 | 0.2900 |

EA, effect allele; OA, other allele; DVT, deep vein thrombosis; EAF, effect allele frequency; SNP, single nucleotide polymorphism; SE, standard error; PE, pulmonary embolism; VTE, venous thromboembolism.
