# Supplementary material for: Genetically predicted basal metabolic rate and venous thromboembolism risk: a Mendelian randomization study
Source: Front Nutr. 2023 Dec 21;10:1263804. doi: 10.3389/fnut.2023.1263804 (PMC10768029; doi:10.3389/fnut.2023.1263804)
Supplement: Supplementary file 12 [file Table_12.DOCX]

Supplementary Table 12 Mendelian randomization analysis evaluating the causal association between VTE and BMR.

| Exposure | MR method | Number of instruments | OR | 95% CI | *P* | MR-Egger intercept | *P* for MR-Egger intercept |
| --- | --- | --- | --- | --- | --- | --- | --- |
| Venous thromboembolism | IVW | 13 | 1.003 | 0.986-1.021 | 0.722 | - | - |
|  | Weighted median | 13 | 1.005 | 0.999-1.012 | 0.078 | - | - |
|  | MR-Egger | 13 | 1.003 | 0.975-1.032 | 0.832 | 6.182×10^-6^ | 0.999 |
| Pulmonary embolism | IVW | 6 | 0.997 | 0.975-1.019 | 0.760 | - | - |
|  | Weighted median | 6 | 1.009 | 0.999-1.019 | 0.068 | - | - |
|  | MR-Egger | 6 | 1.038 | 0.984-1.093 | 0.241 | -0.012 | 0.181 |
| DVT of lower extremities | IVW | 9 | 1.006 | 0.992-1.020 | 0.427 | - | - |
|  | Weighted median | 9 | 1.004 | 1.000-1.008 | 0.052 | - | - |
|  | MR-Egger | 9 | 0.999 | 0.979-1.021 | 0.962 | 0.003 | 0.446 |

BMR, basal metabolic rate; IVW, inverse variance weighted; CI, confidence interval; OR, odds ratio; VTE, venous thromboembolism; DVT, deep vein thrombosis.
